# Supplementary material for: Multi-omic analysis in transgenic mice implicates omega-6/omega-3 fatty acid imbalance as a risk factor for chronic disease
Source: Commun Biol. 2019 Jul 26;2:276. doi: 10.1038/s42003-019-0521-4 (PMC6659714; doi:10.1038/s42003-019-0521-4)
Supplement: Supplementary file 1 — Supplementary Information [file 42003_2019_521_MOESM1_ESM.docx]

**
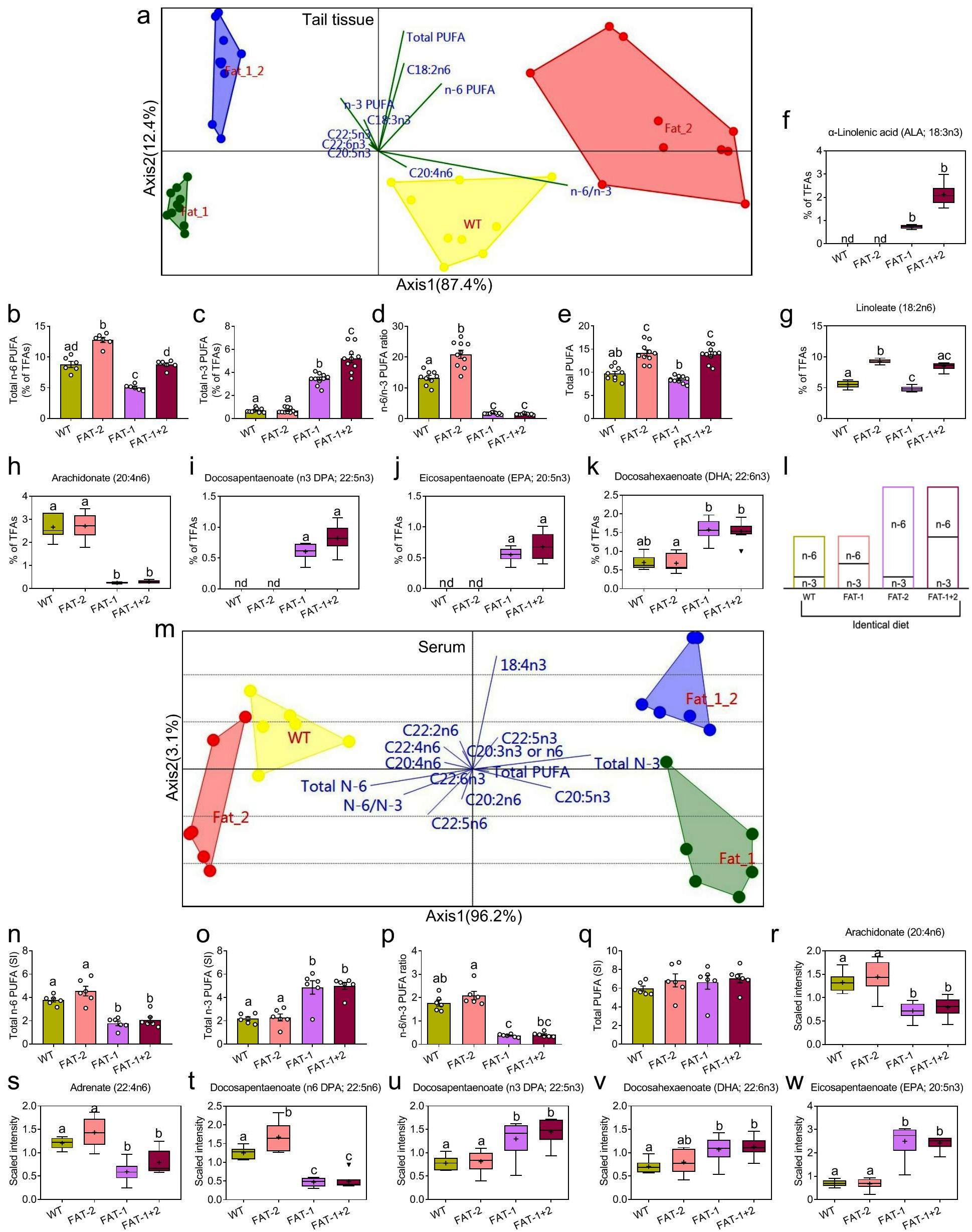
**

**Supplementary Figure 1: Tail and serum polyunsaturated fatty acids (PUFA) profile. (a)** Principal-component analysis (PCA) (variance-covariance type) showing WT (n=9), FAT-2, FAT-1 and FAT-1+2 mice (n=10/group) tail tissue PUFA profile (% of total fatty acids [% of FAs]) including total PUFA and n-6/n-3 PUFA ratio. The magnitude and direction correspond to the weights. (**b-e**) Differences (mean ± SEM) in tail tissue total n-6 PUFA, total n-3 PUFA, n-6/n-3 PUFA ratio and total PUFA between four genotypes. (**f-k**) Box-plots (box showing the mean, median, and the 25th and 75th percentiles, and the whiskers of the graph show the largest and smallest values) showing the abundance of individual PUFAs in the tail tissue. (**l**) Schematic diagram showing proportions of total n-6 and n-3 PUFA (proportionally divided areas of the bars), comparing differences in total PUFA (height of the bars) and n-6/n-3 ratio (ratios between given proportions of n-6 and n-3 PUFA) between four genotypes (WT, FAT-1, FAT-2 and FAT-1+2 respectively) fed identical diet. (**m**) PCA (variance-covariance type) showing serum PUFA profile (scaled intensity [SI]) for WT, FAT-2, FAT-1 and FAT-1+2 mice (n=6/group). (**n-q**) Differences (mean ± SEM) in serum total n-6 PUFA, total n-3 PUFA, n-6/n-3 PUFA ratio and total PUFA (n=6/group). (**r-w**) Box-plots showing the abundance of individual PUFAs in the serum (n=6/group). Data with different superscript letters are significantly different (*P* < 0.05) according to one way ANOVA with Tukey's multiple comparisons test. SEM, standard error of mean.


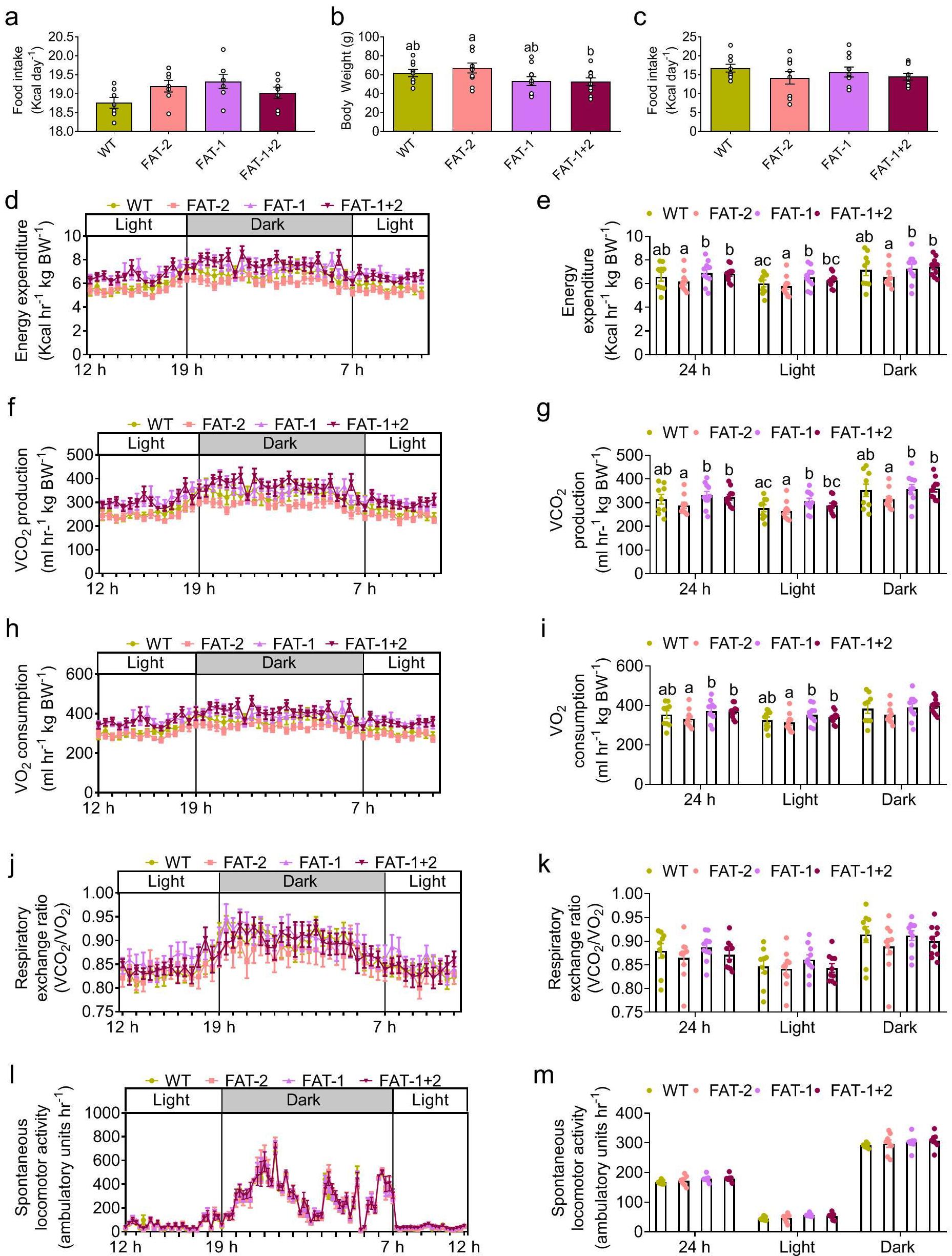


**Supplementary Figure 2**: **Energy metabolism of WT (n = 9), FAT-2, FAT-1 and FAT-1+2 (n = 10/group) transgenic mice. Related to Figure 1**. (**a**) Accumulated Western diet (WD) intake in mice fed ad libitum and average daily food intake (Kcal day^-1^) at the 8th month of WD feeding. (**b**) Body weight (g) over a 16 months’ period. (**c**) Daily WD intake measured in metabolic chambers during indirect calorimetry studies at the 16th month of WD feeding. (**d-e**) Light and dark cycle energy expenditure (Kcal hr^-1^ Kg body weight^-1^) measured in metabolic chambers. (**f-g**) Light and dark cycle of CO2 production (ml hr^-1^ Kg body weight^-1^). (**h-i**) Light and dark cycle of O2 consumption (ml hr^-1^ Kg body weight^-1^). (**j-k**) Respiratory exchange ratio (RER) and average RER in both the light and dark cycles. (**l-m**) Light and dark cycle spontaneous locomotor activities (Ambulatory Counts hr^-1^). Data shown as mean ± SEM. Data with different superscript letters are significantly different (*P* < 0.05) according to ordinary one-way ANOVA (**a-c**, **e**, **g**, **i**, **k**, **m**) or repeated measures two-way ANOVA (**d**, **f**, **h**, **j**, **i**) followed by Tukey's post-hoc test. hr, hour. SEM, standard error of mean.

**
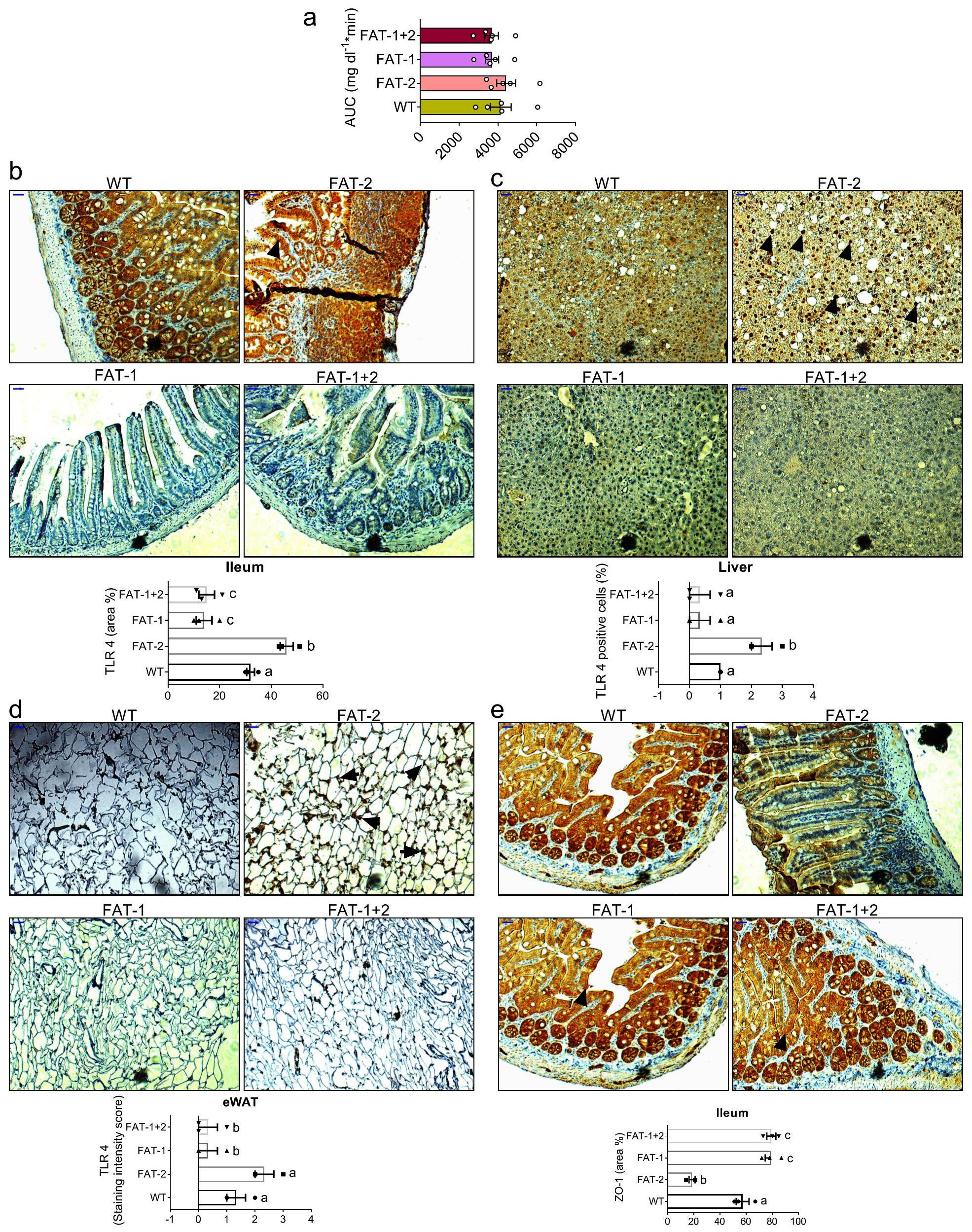
**

**Supplementary Figure 3. Immunohistochemical analyzes. Related to Figure 1.** (a) Area under the curve (AUC) for glucose tolerance test described in Fig. 1f. Representative pictures showing **i**mmunohistochemical (IHC) localization (arrow head) of toll-like receptor 4 (TLR-4) in the ileum (**b**), liver (**c**) and epidydimal white adipose tissue (**d**) and zonulin-1 (ZO-1) (**e**) in the ileum of WT, FAT-2, FAT-1 and FAT-1+2 mice. Images (n=3/group) were analyzed using ImageJ software package and then score (mean ± SEM) were given accordingly. Images were converted from RGB to 8-bit, after which the auto-threshold method of max entropy was applied to all images. Once the threshold was set for each image, the number of pixels of positive staining per field of view (FOV) was counted by using the ‘analyze particles’ function within ImageJ. Data with different superscript letters are significantly different (*P* < 0.05) according to one way ANOVA followed by Tukey’s multiple comparisons test. SEM, standard error of mean. OTU, operational taxonomic unit. Scale bar for immunohistochemical images (**b-e**): 2000µm.

**
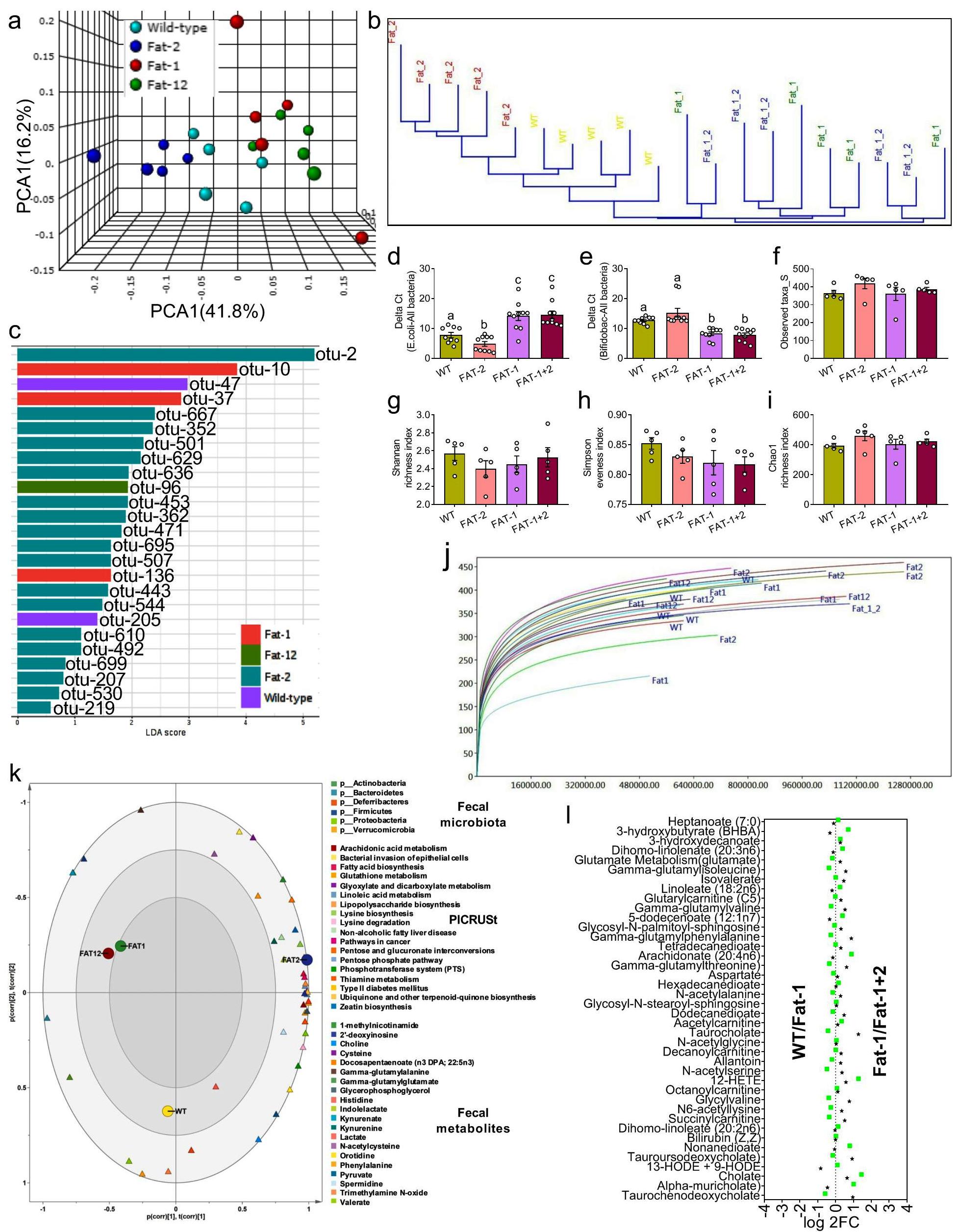
**

**Supplementary Figure 4. Microbiome and metabolome analyses. Related to Figure 2 and 3. (a)** Beta diversity assessment using phylogenetic distance based UniFrac (weighted) analysis and then the results were visualized as Principal coordinate plots (n=5/group). (**b**) Hierarchical clustering (n=5/group) obtained with nonweighted group the average method (unweighted pair-group method with arithmetic means, UPGMA) clustering analysis diagram based on Bray-Curtis distance matrix for four genotypes. Class tree is used to demonstrate similarity between samples, through the clustering tree branch length measure cluster effect. (**c**) Linear discriminant analysis (LDA) scores derived from LEfSe analysis, showing the biomarker taxa (LDA score of >2 and a significance of *P* < 0.05 determined by the Wilcoxon signed-rank test) (n=5/group). (**d-e**) Relative abundance (mean ± SEM) of *family Enterobacteriacea* and *genus Bifidobacterium for* WT (n=9/group), FAT-2, FAT-1 and FAT-1+2 groups (n=10/group). (**f-i**) Dot plots showing α-diversity (within group variations) measures (n=5/group). (**j**) Rarefaction curves (n=5/group). (**k**) Principal-component analysis showing the correlation between differentially expressed fecal bacterial groups (n=5/group), predicted microbial functions (n=5/group) and fecal metabolites (n=6/group) between four genotypes. (**l**) Log 2-fold change (FC) values of serum metabolites (involved in aberrant amino acid biosynthesis, cell turnover regulation, reactive oxygen species neutralization and eicosanoid pathways) obtained with WT vs. FAT-1 and FAT-1 vs. FAT-1+2 pair wise comparisons (n=6/group). Data with different superscript letters are significantly different (*P* < 0.05) according to non-parametric two-sample *t*-test using Monte Carlo permutations (999) (**f**-**i**) or one way ANOVA followed by Tukey’s multiple comparisons test (**d**, **e**) or Welch's two-sample *t*-test (**l**). SEM, standard error of mean.

**
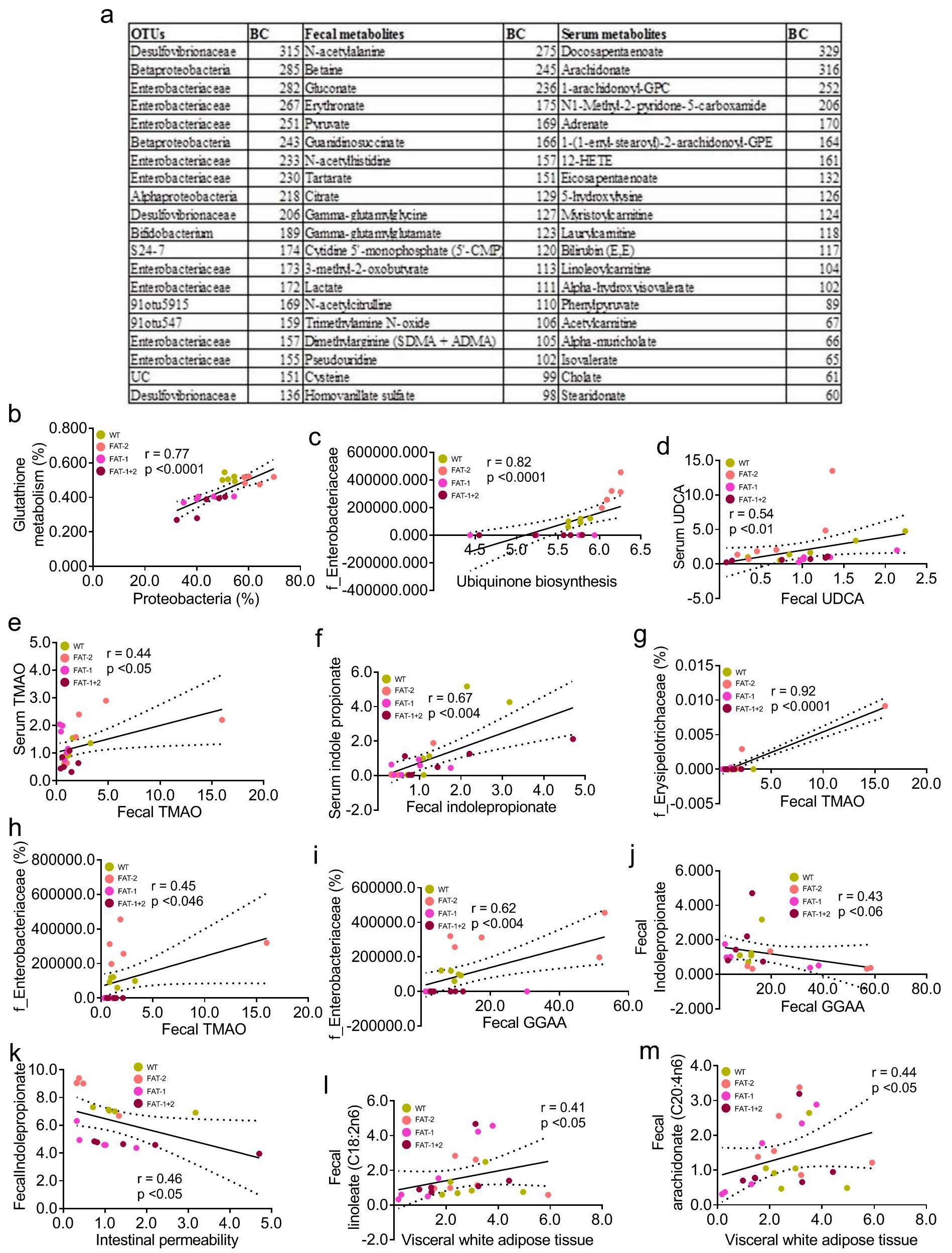
**

**Supplementary Figure 5. Between centrality scores and correlation analyses. Related to Figure 4 and 5.** (**a**) Inter-omic betweeness centrality (BC) scores for differentially expressed fecal microbiota (OTUs), fecal and serum metabolites. **(b-m)** Pearson correlation analysis showing either positive or negative correlations (r values with 95% confidence intervals) between different pairs of data (*P* <0.05 considered significant). The individual values for four genotypes have been grouped using different colors. OTU, operational taxonomic unit. LA, linoleic acid. AA, arachidonic acid. IPA, indolepropionate. GGAA, gamma-glutamyl amino acids. TMAO, trimethylamine N-oxide. UDCA, ursodeoxycholate. WAT, white adipose tissue.

**Supplementary Tables**

**Supplementary Table. 1. KEGG pathways and their association with different disease conditions, Related to Figure 2**

| **Name** | **Association/references** |
| --- | --- |
| Thiamine metabolism | Inflammation^1^ |
| Glutathione metabolism | Inflammation^1^ |
| Zeatin biosynthesis | Inflammation^1^ |
| Arachidonic acid metabolism | Inflammation (colitis)^2^ |
| Linoleic acid metabolism | Inflammation (colon cancer)^3^ |
| Bacterial secretion system | Inflammation^4^ |
| Lipopolysaccharide biosynthesis | Inflammation^5^ |
| Glutathione metabolism | Oxidative stress^5^ |
| Legionellosis | Bacterial translocation^1^ |
| Glutathione metabolism | Bacterial translocation^1^ |
| Lipopolysaccharide biosynthesis | Bacterial translocation^1^ |
| Zeatin biosynthesis | Bacterial translocation^1^ |
| Ubiquinone and other terpenoid quinone biosynthesis | Bacterial translocation^1^ |
| Bacterial invasion of epithelial cells | Bacterial translocation^2^ |
| Vibrio cholerae pathogenic cycle | Bacterial translocation^1^ |
| Salmonella infection | Bacterial translocation^1^ |
| Flagellar assembly | Bacterial translocation^6^ |
| Bacterial chemotaxis | Bacterial translocation^6^ |
| NOD-like receptor signaling pathway | Bacterial translocation^7^ |
| Peptidoglycan biosynthesis | Bacterial translocation^2^ |
| Vibrio cholerae infection | Bacterial translocation^1^ |
| Pathogenic Escherichia coli infection | Bacterial translocation^1^ |
| Bacterial secretion system | Bacterial translocation^4^ |
| Phosphotransferase system (PTS) | Non Alcoholic Fatty Liver Disease^8^ |
| Glycosphingolipid biosynthesis - globo series | Non Alcoholic Fatty Liver Disease^8^ |
| Starch and sucrose metabolism | Non Alcoholic Fatty Liver Disease^8^ |
| Pentose and glucuronate interconversions | Non Alcoholic Fatty Liver Disease^8^ |
| Sulfur metabolism | Non Alcoholic Fatty Liver Disease^9^ |
| Biosynthesis of unsaturated fatty acids | Non Alcoholic Fatty Liver Disease^10^ |
| Lysine degradation | Non Alcoholic Fatty Liver Disease^11^ |
| Fatty acid biosynthesis | Non Alcoholic Fatty Liver Disease^8^ |
| Glyoxylate and dicarboxylate metabolism | Non Alcoholic Fatty Liver Disease^8^ |
| Pentose phosphate pathway | Non Alcoholic Fatty Liver Disease^8^ |
| Lysine biosynthesis | Non Alcoholic Fatty Liver Disease |
| DNA replication | Non Alcoholic Fatty Liver Disease^8^ |
| Taurine and hypotaurine metabolism | Non Alcoholic Fatty Liver Disease^12^ |
| Carbohydrate digestion and absorption | Non Alcoholic Fatty Liver Disease^8^ |
| Cyanoamino acid metabolism | Non Alcoholic Fatty Liver Disease^8^ |
| Non-alcoholic fatty liver disease (NAFLD) | Non Alcoholic Fatty Liver Disease^8^ |
| Type II diabetes mellitus | Metabolic syndrome^13^ |
| Insulin signaling pathway | Metabolic syndrome^14^ |
| Flagellar assembly | Metabolic syndrome^15^ |
| Bacterial chemotaxis | Metabolic syndrome^15^ |
| Type I diabetes mellitus | Metabolism^16^ |
| Pathways in cancer | Cancer^17^ |
| Colorectal cancer | Cancer^17^ |

Predicted microbial genes (those with greatest difference between 4 genotypes) group means from each family selected for FDR corrected *P* < 0.05, obtained with differential abundance analysis.

**Supplementary Table 2. Fecal metabolites (**FDR corrected *P* <0.05) and its association with different disease conditions**, Related to Figure 3**

| **Name** | **Association/reference** |
| --- | --- |
| Docosapentaenoate (DPA; 22:5n3) | Anti-inflammatory^18^ |
| Arachidonate (AA; 20:4n6) | Central obesity^19^ |
| Indolepropionate | Anti-inflammatory/biomarker for the development of T2DM/reduce gut permeability^20-24^ |
| Nicotinate | Anti-inflammatory^25^ |
| 4-methylcatechol sulfate | Apoptosis^26^ |
| Choline | Atherosclerosis^27^ |
| Trimethylamine N-oxide | Atherosclerosis^27,28^ |
| Alanine | Colon cancer^29^ |
| Glutamate | Colon cancer^29^ |
| Valine | Colon cancer^29^ |
| Dimethylglycine | Colon cancer^29^ |
| Proline | Colon cancer^29^ |
| Kynurenate | Colon cancer^30^ |
| 4-hydroxyhippurate | Colon cancer^31,32^ |
| Cholate (major Farnesoid X receptor agonist) | Diabetic lipid phenotype through impaired FXR signaling^33^ |
| Tauro-beta-muricholate (major Farnesoid X receptor Antagonist) | Diabetic lipid phenotype through impaired FXR signaling^33^ |
| 1-methylnicotinamide | Dysbiotic gut microbiota^1^ |
| Trigonelline (N'-methylnicotinate) | Dysbiotic gut microbiota^1^ |
| Spermidine | Dysbiotic gut microbiota^34^ |
| N-acetylcysteine | Increased gut permeability^35^ |
| Heme | Increased gut permeability^35^ |
| Urate | Increased gut permeability^35,36^ |
| Cysteine | Increased gut permeability^35,37^ |
| Gamma-glutamylalanine | Inflammation/Oxidative stress/Glutathione metabolism^38,39^ |
| Gamma-glutamylglutamate | Inflammation/Oxidative stress/Glutathione metabolism^38,39^ |
| Gamma-glutamylglutamine | Inflammation/Oxidative stress/Glutathione metabolism^38,39^ |
| Gamma-glutamylglycine | Inflammation/Oxidative stress/Glutathione metabolism^38,39^ |
| Gamma-glutamylisoleucine | Inflammation/Oxidative stress/Glutathione metabolism^38,39^ |
| Gamma-glutamylmethionine | Inflammation/Oxidative stress/Glutathione metabolism^38,39^ |
| Gamma-glutamylphenylalanine | Inflammation/Oxidative stress/Glutathione metabolism^38,39^ |
| Gamma-glutamylvaline | Inflammation/Oxidative stress/Glutathione metabolism^38,39^ |
| Kynurenine | Insulin resistance^40,41^ |
| Histidine | Intestinal inflammation^42,43^ |
| Kynurenine | Intestinal inflammation^44^ |
| 1-methylhistidine | Intestinal inflammation^43,45^ |
| 1-methylhistamine | Intestinal inflammation/allergic response^43,45,46^ |
| 2'-deoxyuridine | Liver injury^47^ |
| Orotidine | Liver injury^47^ |
| Pseudouridine | Liver injury^47^ |
| Indolelactate | Liver injury^48,49^ |
| 3-indoxyl sulfate | Liver injury^41,48,49^ |
| Valerate | Non-Alcoholic Fatty Liver Disease^11^ |
| Pyruvate | Obesity^48^ |
| Glycerophosphoglycerol | Obesity^48^ |
| Fumarate | Obesity^48^ |
| Malate | Obesity^48^ |
| Lactate | Obesity^29,48^/Production of Reactive Oxygen Species^50^ |
| Succinate | Obesity^29,48,51^ |
| Glycine | Pro-inflammatory^48,51,52^ |
| Glycerophosphoinositol | Pro-inflammatory^53^ |
| Gamma-glutamylalanine | Reduced Colonization resistance to Enterobacteriacea^23^ |
| Gamma-glutamylglutamate | Reduced Colonization resistance to Enterobacteriacea^23^ |
| Gamma-glutamylglutamine | Reduced Colonization resistance to Enterobacteriacea^23^ |
| Gamma-glutamylglycine | Reduced Colonization resistance to Enterobacteriacea^23^ |
| Gamma-glutamylisoleucine | Reduced Colonization resistance to Enterobacteriacea^23^ |
| Gamma-glutamylmethionine | Reduced Colonization resistance to Enterobacteriacea^23^ |
| Gamma-glutamylphenylalanine | Reduced Colonization resistance to Enterobacteriacea^23^ |
| Gamma-glutamylvaline | Reduced Colonization resistance to Enterobacteriacea^23^ |
| Prolylglycine | Reduced Colonization resistance to Enterobacteriacea^23^ |
| Tyrosylglycine | Reduced Colonization resistance to Enterobacteriacea^23^ |
| Methionine | Type 2 DM^54^ |
| Isoleucine | Type 2 DM^29,55^ |
| Leucine | Type 2 DM^29,55^ |
| 2'-deoxyinosine | Type 2 DM^54^ |
| Phenylalanine | Type 2DM^48,54^ |
| P-cresol sulfate | Biomarker of genotoxicity in the colon^56^ |
| P-cresol-glucuronide | Biomarker of genotoxicity in the colon^56^ |

**Supplementary Table. 3. Serum metabolites (**FDR corrected *P* <0.05) and its association with different disease conditions**, Related to Figure 3**

| **Name** | **Association/reference** |
| --- | --- |
| Orotidine | Liver injury^23^ |
| 1-docosahexaenoylglycerol (22:6) | Anti-inflammatory^57^ |
| Eicosapentaenoate (EPA; 20:5n3) | Anti-inflammatory^58^ |
| Alpha-CEHC | Anti-oxidant^59^ |
| Trimethylamine N-oxide/Choline | Atherosclerosis^27^ |
| Isovalerate | Obesity and metabolic syndrome^60^ |
| Isovalerylcarnitine | Abdominal adiposity^61^ |
| 3-hydroxysebacate | Colon cancer^62^ |
| Arabitol/Xylitol | Cancer^63^ |
| 12-HETE (n-6 PUFA-derived oxidized lipid) | Chronic low grade inflammation/NASH/HCC^64-66^ |
| Corticosterone | Epididymal adiposity/ROS production ^67^ |
| Dodecanedioate/Hexadecanedioate | Metabolic syndrome^68^ |
| Tetradecanedioate/ Octadecanedioate | Metabolic syndrome^68^ |
| 15-HETE (n-6 PUFA-derived oxidized lipid) | HCC^66^ |
| Glutarylcarnitine (C5) | HCC^66^ |
| Acetylcarnitine | HCC^66^ |
| Succinylcarnitine | HCC^66^ |
| 13-HODE + 9-HODE (15-LOX products of n-6 linoleic acid) | HCC^66^ |
| Arachidonate (20:4n6) | HCC^66^ |
| Linoleate (18:2n6) | HCC^66^ |
| Linolenate [alpha or gamma; (18:3n3 or 6)] | HCC^66^ |
| Dihomo-linolenate (20:3n3 or n6) | HCC^66^ |
| Bilirubin (Z,Z) | Development of metabolic syndrome^69,70^ |
| Bilirubin (E,E) | Impaired glucose tolerance and type 2 diabetes/Elevated inflammation^41,69,70^ |
| Acetylcarnitine | Insulin resistance (significant positive correlation with HbA_1c_ levels)^55,71^ |
| Alpha-hydroxyisovalerate | Insulin resistance (greater adiposity and blood pressure and dietary-induced NAFLD)^72-74^ |
| Cis-4-decenoyl carnitine | Insulin resistance^71,75^ |
| Laurylcarnitine | Insulin resistance^71,75^ |
| Linoleoylcarnitine | Insulin resistance^71,75^ |
| Myristoylcarnitine | Insulin resistance^71,75^ |
| 3-hydroxyisobutyrate | Insulin resistance^76^ |
| Thromboxane B2 | Metabolic syndrome^77^ |
| N-acetylasparagine | Metabolically unhealthy centrally obese^78^ |
| Adenosine 5'-monophosphate (AMP) | Metabolic-related diseases^79^ |
| Docosapentaenoate (n6 DPA; 22:5n6) | NAFLD^80^ |
| Alpha-muricholate | NAFLD/Hepatocellular carcinoma^81,82^ |
| Cholate | NAFLD/Hepatocellular carcinoma^81,82^ |
| Glutarylcarnitine (C5) | Obesity^71,75^ |
| N6-acetyllysine | Obesity^71,75^ |
| Phenylpyruvate | Obesity^83^ |
| 1-(1-enyl-stearoyl)-2-arachidonoyl-GPE (P-18:0/20:4)* | Onset of vascular inflammation and initiation of atherosclerosis^84^ |
| Allantoin | Oxidative stress^85^ |
| Stearidonate (18:4n3) | Oxidative stress and inflammation^86^ |
| 12,13-DiHOME | Postprandial inflammation^87^ |
| 3-methylglutaconate | Pre-diabetes and diabetes^88^ |
| 1-arachidonoyl-GPC (20:4n6) | Pro-inflammatory^89,90^ |
| Adrenate (22:4n6) | Pro-inflammatory^91^ |
| 13-HODE + 9-HODE (15-LOX products of n-6 linoleic acid) | Pro-inflammatory/Oxidative stress/PPAR-α agonism induced hepatic micro vesicular steatosis^91-95^ |
| 5-hydroxylysine | Protein oxidation^96^ |
| 6-oxopiperidine-2-carboxylic acid | Protein oxidation^96^ |
| 3-methylhistidine | Skeletal muscle protein turnover ^68^ |
| Ursodeoxycholate | Stimulation of hepatocyte proliferation and tumor growth/ Atherosclerosis^97,98^ |
| 2-aminooctanoate | Type 2DM^99^ |
| Malonylcarnitine | Type 2DM^100^ |
| Glucose | Type 2DM^101^ |
| Adenosine 5'-monophosphate (AMP) | Type 2DM^101^ |
| Indolepropionate | Type 2DM^22^ |
| Glycerol | Type 2DM^101^ |
| Glycerophosphorylcholine (GPC) | Type 2DM^101^ |
| Phosphate | Type 2DM^101^ |
| Hypoxanthine | Type 2DM^101^ |
| N1-Methyl-2-pyridone-5-carboxamide | Up-regulation of mitochondrial oxidative pathways and associated obesity^102^ |
| Arachidonate (20:4n6) | Visceral fat accumulation/Pro-inflammatory/oxidative stress ^103-105^ |
| Adrenate (22:4n6) | NAFLD^106^ |

**Supplementary Table 4**. **Fatty acid composition of Western diet (AIN-76A-1816187-200) used in this study**.

| **Fatty acid (FA)** | **% of total FAs** |
| --- | --- |
| C8:0 (Caprylic acid) | 2.04 |
| C10:0 (Capric acid) | 16.68 |
| C12:0 (Lauric acid) | 8.32 |
| C14:0 (Myristic acid) | 0.39 |
| C14:1(7-Tetradecenoic acid) | 0.34 |
| C16:0 (Palmitic acid) | 20.02 |
| C16:1(Palmitoleic acid) | 1.94 |
| C17:0 (Margaric acid | 0.84 |
| C17:1(Heptadecenoic acid) | 0.00 |
| C18:0 (Stearic acid) | 11.97 |
| C18:1(Vaccenic acid) | 35.19 |
| C18:2 (n6) (Linoleic acid) | 1.57 |
| C18:3 (n6) (γ-Linolenic acid) | 0.06 |
| C18:3 (n3) (α-Linolenic acid) | 0.08 |
| C20:0 (Arachidic acid) | 0.11 |
| C20:1(Paullinic acid) | 0.37 |
| C20:2 (n6) (Eicosadienoic acid) | 0.02 |
| C20:3 (n6) (Dihomo-γ-linolenic acid) | 0.00 |
| C20:4 (n6) (Arachidonic acid) | 0.00 |
| C20:3 (n3) (Eicosatrienoic acid) | 0.00 |
| C20:5 (Eicosapentaenoic acid) | 0.00 |
| C22:0 (Behenic acid) | 0.04 |
| C22:1(15-Docosenoic acid) | 0.04 |
| C22:2 (n6) (Docosadienoic acid) | 0.00 |
| C22:4 (n6) (Adrenic acid) | 0.00 |
| C22:5 (Docosapentaenoic acid) | 0.00 |
| C22:6 (Docosahexaenoic acid) | 0.00 |
| C24:0 (Lignoceric acid) | 0.00 |
| C24:1(17-Tetracosenoic acid) | 0.00 |
| Saturated FA (SFA) | 60.39 |
| Monounsaturated FA (MUFA) | 37.88 |
| Polyunsaturated FA (PUFA) | 1.73 |
| n-6 PUFA | 1.65 |
| n-3 PUFA | 0.08 |
| n-6/n-3 PUFA ratio | 20.62 |

**Supplementary Table 5. Oligonucleotides used for this study.**

| **Name** | **Sequence** |
| --- | --- |
| All bacteria.fwd | \| ACTCCTACGGGAGGCAGCAGT \| \| --- \| |
| All bacteria.rev | ATTACCGCGGCTGCTGGC |
| Enterobacteriacea.fwd | \| GTGCCAGCAGCCGCGGTAA \| \| --- \| |
| Enterobacteriacea.rev | GCCTCAAGGGCACAACCTCCAAG |
| Escherichia Coli.fwd | CATGCCGCGTGTATGAAGAA |
| Escherichia Coli.rev | CGGGTAACGTCAATGAGCAAA |
| Bifidobacterium.fwd | CGGGTGAGTAATGCGTGACC |
| Bifidobacterium.rev | TGATAGGACGCGACCCCA |

**Supplementary Methods**

**Host-microbiome interaction analyses**

*a) RV coefficient*

To assesses the overall association between the microbial (FDR-corrected *P* value < 0.05) and host parameters (serum total PUFA and n-6 and n-3 PUFA, n-6/n-3 ratio, body weight, HCC incidence, IP, serum LPS, LBP, TNF-α and IL-6), the RV coefficient (multivariate generalization of the Pearson correlation coefficient)^107^ was calculated using the XLSTAT software version 2017.6.

b) *Correlation network analysis*

The complex host-microbe interactions can be extricated by network-based analytical approaches as described previously^107-109^. Host-microbiota interaction network was built from Spearman’s non-parametric rank correlation coefficient (*P* < 0.05) between host parameters (serum total PUFA and n-6 and n-3 PUFA, n-6/n-3 ratio, body weight, HCC incidence, IP, serum LPS, LBP, TNF-α and IL-6) and microbial and metabolite parameters with false discovery rate-corrected *P* values < 0.05. Nodes (filled circles) represents host parameters (cyan), microbes (pink), fecal (green) and serum (olive) metabolites. Node size reflects betweeness centrality— a measure of how many shortest paths within the entire network passes through the node in question (crucial to the communication within the network). Lines (edges) represent statistically significant correlations (*P* <0.05) and are colored grey for positive and blue for negative correlations. The PAST software version 2.17 was used to calculate the correlations. Gephi Graph Visualization and Manipulation software version 0.9.2^107^ was used to visualize the network. The highly central or hubs are the nodes with the highest betweeness centrality in the network. A “modularity” community detection algorithm was utilized to separate the first 4 largest modules. A module in the network is a set of nodes connected to each other by many links, while connected by few links to nodes of other groups. It is important to note that the modules are elementary units of any biological network, and their identification and characterization provides us with more information about the local interaction patterns in the network and their contribution to the overall structure, connectivity, and function of the network. When considered as isolated, taxonomic, evolutionary, or functional modules, modules are biologically important. High modularity shows that the network has dense connections within certain groups of nodes and spars connections between these groups.

*c) Partial Least Square-Regression (PLS-R)*

To assesses the association between microbial and host parameters, the partial least square regression (PLS-R), including jackknife-based variable selection, was also used as described previously^107,109^. Because the number of explanatory variables were high, and they were probably correlated, the PLS-R with leave one-out cross-validation (LOO-CV) method was used. The global goodness of fit and the predictive quality of the models were proved by the Q2 cumulated index (Q2_cum_) measures. To test the validity of the model against over-fitting, the Q2_cum_ was used. The cumulated R^2^Y and R^2^X measures were very close to one with two components in the model. This shows that the two components generated by the PLR-R summarize well both the Xs and the Ys. PLS scatter plots for subject clustering and variables were used to present the results. The R^2^ (coefficient of determination) shows the % of variability of the dependent variable (Y) which is explained by the explanatory variables (X). The corresponding identified modules (Fig. 5b–e) in the correlation networks were compared with the parameters (variable importance in the projection values 1 or > 1.0) contributing to the multivariate PLS models. All analyses were accomplished using the XLSTAT software version 2017.6.

**Multiomics integrative analyses**

*a) Multiple factor analysis*

As described previously^109^, the multiple factor analysis was utilized to asesses the associations between the microbiome and fecal and serum metabolome data sets. Multiple factor analysis using Spearman type principal component analysis was performed to superimpose the microbiome and fecal and serum metabolites data. In the first part of the analysis, a Spearman type principal component analysis (PCA) successively carried out for each dataset which stores the value of the first eigenvalue of each analysis to then weigh the various datasets in the second part of the analysis, where the weighted PCA on the columns of all the datasets leads to each indicator variable having a weight that is a function of the frequency of the corresponding category. Then, the coordinates of the projected points in the space resulting from the multiple factor analysis are displayed. The projected points correspond to projections of the observations in the spaces reduced to the dimensions of each dataset. Based on the eigenvalues of the weighted PCA, the first two factors (F1/F2) covered the variability in this analysis. To test whether the two groups with superimposed microbiome and metabolome data were separated from each other, Mann-Whitney testing was performed on the coordinates of the projected points of all the observations and p values were obtained using 10,000 Monte Carlo simulations. One end of each line for an observation indicates the metabolomics data (differently colored to indicate the groups) and another end (red) indicates the microbiota. All analyses were accomplished using the XLSTAT software version 2017.6.

*b)* [*Partial Least Squares Discriminant Analysis*](https://www.xlstat.com/en/solutions/features/pls-discriminant-analysis) *(PLS-DA)*

Multiomics integration was completed using XLSTAT software version 2017.6. A PLS-DA model [Balanced n-6/n-3 PUFA ratio (FAT-1 and FAT-1+2 samples) to imbalanced n-6/n-3 PUFA ratio (wild-type and FAT-2 samples)] was built to study the three combined data sets (16s rRNA gene sequencing and fecal and serum metabolomics data) as described previously. For integration of all 3 different omics data sets, the samples were aligned in one matrix and the data was rescaled from 0 to 1. PLS-DA loading plots were constructed to simultaneously visualize features of the microbiota and fecal and serum metabolome impacted by balanced and imbalanced n-6/n-3 PUFA ratios. The model was validated by the jackknife leave one-out cross-validation method. The global goodness of fit and the predictive quality of the models were proved by the Q2 cumulated index (Q2_cum_) measures. To test the validity of the model against over-fitting, the Q2_cum_ was used. The cumulated R^2^Y and R^2^X measures were very close to one with two components in the model. This shows that the two components generated by the PLR-R summarize well both the Xs and the Ys.

*c) Correlation network analysis*

To investigate the individual microbe–metabolite correlations that support the relationship between microbiota and metabolites, an inter’omic network was constructed using all three data sets (16s sequencing and fecal and serum metabolites data with FDR corrected P < 0.05) as described previously^107-109^. Spearman’s non-parametric rank correlation coefficients were calculated for all microbe-microbe, metabolite–metabolite, and microbe–metabolite pairs using the PAST software version 2.17. Gephi Graph Visualization and Manipulation software version 0.9.2^107^ was used to visualize the network. The nodes (filled circles) represented the microbes (pink) and fecal (green) and serum (olive) metabolites. The node size reflected the inter-omic betweeness centrality— a measure of how many shortest paths within the entire network passes through the node in question (crucial to the communication within the network). The names of the selected microbes and metabolites with higher inter-omic degree centrality—the number of connections to nodes of the opposite data type (i.e., microbe–metabolite pairs) were shown to improve the clarity of the network. The edges represented the statistically significant (spearman’s non-parametric rank correlation coefficient) positive (grey) and negative (blue) correlations (p < 0.05) between microbe–microbe, metabolite–metabolite, or microbe–metabolite pairs. The highly central or hubs are the nodes with the highest betweeness centrality in the network. A “modularity” community detection algorithm was utilized to separate the first 3 largest modules. A module in the network is a set of nodes connected to each other by many links, while connected by few links to nodes of other groups. It is important to note that the modules are elementary units of any biological network, and their identification and characterization provides us with more information about the local interaction patterns in the network and their contribution to the overall structure, connectivity, and function of the network. When considered as isolated, taxonomic, evolutionary, or functional modules, modules are biologically important. High modularity shows that the network has dense connections within certain groups of nodes and spars connections between these groups.

**Supplementary References**

1 Vazquez-Castellanos, J. F. *et al.* Altered metabolism of gut microbiota contributes to chronic immune activation in HIV-infected individuals. *Mucosal Immunol* **8**, 760-772, doi:10.1038/mi.2014.107 (2015).

2 Munyaka, P. M., Eissa, N., Bernstein, C. N., Khafipour, E. & Ghia, J. E. Antepartum Antibiotic Treatment Increases Offspring Susceptibility to Experimental Colitis: A Role of the Gut Microbiota. *PLoS One* **10**, e0142536, doi:10.1371/journal.pone.0142536 (2015).

3 Cui, M. *et al.* Gene expression analysis of colorectal cancer by bioinformatics strategy. *Hepatogastroenterology* **61**, 1942-1945 (2014).

4 Keshavarzian, A. *et al.* Colonic bacterial composition in Parkinson's disease. *Mov Disord* **30**, 1351-1360, doi:10.1002/mds.26307 (2015).

5 Pinheiro de Oliveira, F. *et al.* Phenylketonuria and Gut Microbiota: A Controlled Study Based on Next-Generation Sequencing. *PLoS One* **11**, e0157513, doi:10.1371/journal.pone.0157513 (2016).

6 McFadden, R. M. *et al.* The Role of Curcumin in Modulating Colonic Microbiota During Colitis and Colon Cancer Prevention. *Inflamm Bowel Dis* **21**, 2483-2494, doi:10.1097/MIB.0000000000000522 (2015).

7 Franchi, L., Warner, N., Viani, K. & Nunez, G. Function of Nod-like receptors in microbial recognition and host defense. *Immunol Rev* **227**, 106-128, doi:10.1111/j.1600-065X.2008.00734.x (2009).

8 Boursier, J. *et al.* The severity of nonalcoholic fatty liver disease is associated with gut dysbiosis and shift in the metabolic function of the gut microbiota. *Hepatology* **63**, 764-775, doi:10.1002/hep.28356 (2016).

9 Toohey, J. I. Sulfur amino acids in diet-induced fatty liver: a new perspective based on recent findings. *Molecules* **19**, 8334-8349, doi:10.3390/molecules19068334 (2014).

10 Ni, Y. *et al.* Circulating Unsaturated Fatty Acids Delineate the Metabolic Status of Obese Individuals. *EBioMedicine* **2**, 1513-1522, doi:10.1016/j.ebiom.2015.09.004 (2015).

11 Michail, S. *et al.* Altered gut microbial energy and metabolism in children with non-alcoholic fatty liver disease. *FEMS Microbiol Ecol* **91**, 1-9, doi:10.1093/femsec/fiu002 (2015).

12 Liu, S. *et al.* Interaction of genotype and diet on small intestine microbiota of Japanese quail fed a cholesterol enriched diet. *Sci Rep* **8**, 2381, doi:10.1038/s41598-018-20508-9 (2018).

13 Grasset, E. *et al.* A Specific Gut Microbiota Dysbiosis of Type 2 Diabetic Mice Induces GLP-1 Resistance through an Enteric NO-Dependent and Gut-Brain Axis Mechanism. *Cell Metab* **25**, 1075-1090 e1075, doi:10.1016/j.cmet.2017.04.013 (2017).

14 Inoue, R. *et al.* Prediction of functional profiles of gut microbiota from 16S rRNA metagenomic data provides a more robust evaluation of gut dysbiosis occurring in Japanese type 2 diabetic patients. *J Clin Biochem Nutr* **61**, 217-221, doi:10.3164/jcbn.17-44 (2017).

15 Louis, S., Tappu, R. M., Damms-Machado, A., Huson, D. H. & Bischoff, S. C. Characterization of the Gut Microbial Community of Obese Patients Following a Weight-Loss Intervention Using Whole Metagenome Shotgun Sequencing. *PLoS One* **11**, e0149564, doi:10.1371/journal.pone.0149564 (2016).

16 Leiva-Gea, I. *et al.* Gut Microbiota Differs in Composition and Functionality Between Children With Type 1 Diabetes and MODY2 and Healthy Control Subjects: A Case-Control Study. *Diabetes Care* **41**, 2385-2395, doi:10.2337/dc18-0253 (2018).

17 Zeller, G. *et al.* Potential of fecal microbiota for early-stage detection of colorectal cancer. *Mol Syst Biol* **10**, 766, doi:10.15252/msb.20145645 (2014).

18 Kaliannan, K., Wang, B., Li, X. Y., Kim, K. J. & Kang, J. X. A host-microbiome interaction mediates the opposing effects of omega-6 and omega-3 fatty acids on metabolic endotoxemia. *Sci Rep* **5**, 11276, doi:10.1038/srep11276 (2015).

19 Zierer, J. *et al.* The fecal metabolome as a functional readout of the gut microbiome. *Nat Genet* **50**, 790-795, doi:10.1038/s41588-018-0135-7 (2018).

20 Tuomainen, M. *et al.* Associations of serum indolepropionic acid, a gut microbiota metabolite, with type 2 diabetes and low-grade inflammation in high-risk individuals. *Nutr Diabetes* **8**, 35, doi:10.1038/s41387-018-0046-9 (2018).

21 Jennis, M. *et al.* Microbiota-derived tryptophan indoles increase after gastric bypass surgery and reduce intestinal permeability in vitro and in vivo. *Neurogastroenterol Motil* **30**, doi:10.1111/nmo.13178 (2018).

22 de Mello, V. D. *et al.* Indolepropionic acid and novel lipid metabolites are associated with a lower risk of type 2 diabetes in the Finnish Diabetes Prevention Study. *Sci Rep* **7**, 46337, doi:10.1038/srep46337 (2017).

23 Jump, R. L. *et al.* Metabolomics analysis identifies intestinal microbiota-derived biomarkers of colonization resistance in clindamycin-treated mice. *PLoS One* **9**, e101267, doi:10.1371/journal.pone.0101267 (2014).

24 Fiorucci, S. & Distrutti, E. Bile Acid-Activated Receptors, Intestinal Microbiota, and the Treatment of Metabolic Disorders. *Trends Mol Med* **21**, 702-714, doi:10.1016/j.molmed.2015.09.001 (2015).

25 Singh, N. *et al.* Activation of Gpr109a, receptor for niacin and the commensal metabolite butyrate, suppresses colonic inflammation and carcinogenesis. *Immunity* **40**, 128-139, doi:10.1016/j.immuni.2013.12.007 (2014).

26 Pan, P. *et al.* Beneficial Regulation of Metabolic Profiles by Black Raspberries in Human Colorectal Cancer Patients. *Cancer Prev Res (Phila)* **8**, 743-750, doi:10.1158/1940-6207.CAPR-15-0065 (2015).

27 Wang, Z. *et al.* Gut flora metabolism of phosphatidylcholine promotes cardiovascular disease. *Nature* **472**, 57-63, doi:10.1038/nature09922 (2011).

28 Oellgaard, J., Winther, S. A., Hansen, T. S., Rossing, P. & von Scholten, B. J. Trimethylamine N-oxide (TMAO) as a New Potential Therapeutic Target for Insulin Resistance and Cancer. *Curr Pharm Des* **23**, 3699-3712, doi:10.2174/1381612823666170622095324 (2017).

29 Lin, Y. *et al.* NMR-based fecal metabolomics fingerprinting as predictors of earlier diagnosis in patients with colorectal cancer. *Oncotarget* **7**, 29454-29464, doi:10.18632/oncotarget.8762 (2016).

30 Rombouts, C. *et al.* Untargeted metabolomics of colonic digests reveals kynurenine pathway metabolites, dityrosine and 3-dehydroxycarnitine as red versus white meat discriminating metabolites. *Sci Rep* **7**, 42514, doi:10.1038/srep42514 (2017).

31 He, X., Ji, G., Jia, W. & Li, H. Gut Microbiota and Nonalcoholic Fatty Liver Disease: Insights on Mechanism and Application of Metabolomics. *Int J Mol Sci* **17**, 300, doi:10.3390/ijms17030300 (2016).

32 Zhang, A. *et al.* Metabolomics in diagnosis and biomarker discovery of colorectal cancer. *Cancer Lett* **345**, 17-20, doi:10.1016/j.canlet.2013.11.011 (2014).

33 Duparc, T. *et al.* Hepatocyte MyD88 affects bile acids, gut microbiota and metabolome contributing to regulate glucose and lipid metabolism. *Gut* **66**, 620-632, doi:10.1136/gutjnl-2015-310904 (2017).

34 Levy, M. *et al.* Microbiota-Modulated Metabolites Shape the Intestinal Microenvironment by Regulating NLRP6 Inflammasome Signaling. *Cell* **163**, 1428-1443, doi:10.1016/j.cell.2015.10.048 (2015).

35 Ijssennagger, N. *et al.* Gut microbiota facilitates dietary heme-induced epithelial hyperproliferation by opening the mucus barrier in colon. *Proc Natl Acad Sci U S A* **112**, 10038-10043, doi:10.1073/pnas.1507645112 (2015).

36 Quifer-Rada, P., Choy, Y. Y., Calvert, C. C., Waterhouse, A. L. & Lamuela-Raventos, R. M. Use of metabolomics and lipidomics to evaluate the hypocholestreolemic effect of Proanthocyanidins from grape seed in a pig model. *Mol Nutr Food Res* **60**, 2219-2227, doi:10.1002/mnfr.201600190 (2016).

37 Blachier, F., Beaumont, M. & Kim, E. Cysteine-derived hydrogen sulfide and gut health: a matter of endogenous or bacterial origin. *Curr Opin Clin Nutr Metab Care*, doi:10.1097/MCO.0000000000000526 (2018).

38 Zheng, Y., Yu, B., Alexander, D., Steffen, L. M. & Boerwinkle, E. Human metabolome associates with dietary intake habits among African Americans in the atherosclerosis risk in communities study. *Am J Epidemiol* **179**, 1424-1433, doi:10.1093/aje/kwu073 (2014).

39 Preidis, G. A. *et al.* The undernourished neonatal mouse metabolome reveals evidence of liver and biliary dysfunction, inflammation, and oxidative stress. *J Nutr* **144**, 273-281, doi:10.3945/jn.113.183731 (2014).

40 Favennec, M. *et al.* The kynurenine pathway is activated in human obesity and shifted toward kynurenine monooxygenase activation. *Obesity (Silver Spring)* **23**, 2066-2074, doi:10.1002/oby.21199 (2015).

41 Lustgarten, M. S. & Fielding, R. A. Metabolites Associated With Circulating Interleukin-6 in Older Adults. *J Gerontol A Biol Sci Med Sci* **72**, 1277-1283, doi:10.1093/gerona/glw039 (2017).

42 Calvani, R. *et al.* Fecal and urinary NMR-based metabolomics unveil an aging signature in mice. *Exp Gerontol* **49**, 5-11, doi:10.1016/j.exger.2013.10.010 (2014).

43 Derakhshani, H. *et al.* The Features of Fecal and Ileal Mucosa-Associated Microbiota in Dairy Calves during Early Infection with Mycobacterium avium Subspecies paratuberculosis. *Front Microbiol* **7**, 426, doi:10.3389/fmicb.2016.00426 (2016).

44 Morris, G. *et al.* The Role of the Microbial Metabolites Including Tryptophan Catabolites and Short Chain Fatty Acids in the Pathophysiology of Immune-Inflammatory and Neuroimmune Disease. *Mol Neurobiol* **54**, 4432-4451, doi:10.1007/s12035-016-0004-2 (2017).

45 Yang, D. & Hong, J. H. Dexmedetomidine Modulates Histamine-induced Ca(2+) Signaling and Pro-inflammatory Cytokine Expression. *Korean J Physiol Pharmacol* **19**, 413-420, doi:10.4196/kjpp.2015.19.5.413 (2015).

46 Shankar, V. *et al.* Differences in Gut Metabolites and Microbial Composition and Functions between Egyptian and U.S. Children Are Consistent with Their Diets. *mSystems* **2**, doi:10.1128/mSystems.00169-16 (2017).

47 P, O. B. *et al.* Intestinal microbiota-derived metabolomic blood plasma markers for prior radiation injury. *Int J Radiat Oncol Biol Phys* **91**, 360-367, doi:10.1016/j.ijrobp.2014.10.023 (2015).

48 Phetcharaburanin, J. *et al.* Systemic Characterization of an Obese Phenotype in the Zucker Rat Model Defining Metabolic Axes of Energy Metabolism and Host-Microbial Interactions. *J Proteome Res* **15**, 1897-1906, doi:10.1021/acs.jproteome.6b00090 (2016).

49 Kurland, I. J. *et al.* Integrative Metabolic Signatures for Hepatic Radiation Injury. *PLoS One* **10**, e0124795, doi:10.1371/journal.pone.0124795 (2015).

50 Iatsenko, I., Boquete, J. P. & Lemaitre, B. Microbiota-Derived Lactate Activates Production of Reactive Oxygen Species by the Intestinal NADPH Oxidase Nox and Shortens Drosophila Lifespan. *Immunity* **49**, 929-942 e925, doi:10.1016/j.immuni.2018.09.017 (2018).

51 Curtis, M. M. *et al.* The gut commensal Bacteroides thetaiotaomicron exacerbates enteric infection through modification of the metabolic landscape. *Cell Host Microbe* **16**, 759-769, doi:10.1016/j.chom.2014.11.005 (2014).

52 Carmans, S. *et al.* The inhibitory neurotransmitter glycine modulates macrophage activity by activation of neutral amino acid transporters. *J Neurosci Res* **88**, 2420-2430, doi:10.1002/jnr.22395 (2010).

53 Corda, D., Zizza, P., Varone, A., Filippi, B. M. & Mariggio, S. The glycerophosphoinositols: cellular metabolism and biological functions. *Cell Mol Life Sci* **66**, 3449-3467, doi:10.1007/s00018-009-0113-4 (2009).

54 Zhu, Y. *et al.* Fecal metabonomic study of a polysaccharide, MDG-1 from Ophiopogon japonicus on diabetic mice based on gas chromatography/time-of-flight mass spectrometry (GC TOF/MS). *Mol Biosyst* **10**, 304-312, doi:10.1039/c3mb70392d (2014).

55 Schooneman, M. G., Vaz, F. M., Houten, S. M. & Soeters, M. R. Acylcarnitines: reflecting or inflicting insulin resistance? *Diabetes* **62**, 1-8, doi:10.2337/db12-0466 (2013).

56 Al Hinai, E. A. *et al.* Modelling the role of microbial p-cresol in colorectal genotoxicity. *Gut Microbes*, 1-14, doi:10.1080/19490976.2018.1534514 (2018).

57 Budhu, A. *et al.* Metabolic profiles are principally different between cancers of the liver, pancreas and breast. *Int J Biol Sci* **10**, 966-972, doi:10.7150/ijbs.9810 (2014).

58 Pischon, T. *et al.* Habitual dietary intake of n-3 and n-6 fatty acids in relation to inflammatory markers among US men and women. *Circulation* **108**, 155-160, doi:10.1161/01.CIR.0000079224.46084.C2 (2003).

59 Wefers, H. & Sies, H. The protection by ascorbate and glutathione against microsomal lipid peroxidation is dependent on vitamin E. *Eur J Biochem* **174**, 353-357 (1988).

60 Ryan, P. M. *et al.* Microbiome and metabolome modifying effects of several cardiovascular disease interventions in apo-E(-/-) mice. *Microbiome* **5**, 30, doi:10.1186/s40168-017-0246-x (2017).

61 Lustgarten, M. S., Price, L. L., Phillips, E. M. & Fielding, R. A. Serum glycine is associated with regional body fat and insulin resistance in functionally-limited older adults. *PLoS One* **8**, e84034, doi:10.1371/journal.pone.0084034 (2013).

62 Brown, D. G., Borresen, E. C., Brown, R. J. & Ryan, E. P. Heat-stabilised rice bran consumption by colorectal cancer survivors modulates stool metabolite profiles and metabolic networks: a randomised controlled trial. *Br J Nutr* **117**, 1244-1256, doi:10.1017/S0007114517001106 (2017).

63 Cheng, Y. *et al.* Expanded metabolomics approach to profiling endogenous carbohydrates in the serum of ovarian cancer patients. *J Sep Sci* **39**, 316-323, doi:10.1002/jssc.201500964 (2016).

64 Moller, K. *et al.* Influence of weight reduction on blood levels of C-reactive protein, tumor necrosis factor-alpha, interleukin-6, and oxylipins in obese subjects. *Prostaglandins Leukot Essent Fatty Acids* **106**, 39-49, doi:10.1016/j.plefa.2015.12.001 (2016).

65 Depner, C. M. *et al.* A metabolomic analysis of omega-3 fatty acid-mediated attenuation of western diet-induced nonalcoholic steatohepatitis in LDLR-/- mice. *PLoS One* **8**, e83756, doi:10.1371/journal.pone.0083756 (2013).

66 Fitian, A. I. *et al.* Integrated metabolomic profiling of hepatocellular carcinoma in hepatitis C cirrhosis through GC/MS and UPLC/MS-MS. *Liver Int* **34**, 1428-1444, doi:10.1111/liv.12541 (2014).

67 Yu, J. *et al.* Chronic glucocorticoid exposure-induced epididymal adiposity is associated with mitochondrial dysfunction in white adipose tissue of male C57BL/6J mice. *PLoS One* **9**, e112628, doi:10.1371/journal.pone.0112628 (2014).

68 Korsholm, A. S., Kjaer, T. N., Ornstrup, M. J. & Pedersen, S. B. Comprehensive Metabolomic Analysis in Blood, Urine, Fat, and Muscle in Men with Metabolic Syndrome: A Randomized, Placebo-Controlled Clinical Trial on the Effects of Resveratrol after Four Months' Treatment. *Int J Mol Sci* **18**, doi:10.3390/ijms18030554 (2017).

69 Lee, Y. B. *et al.* Change in Serum Bilirubin Level as a Predictor of Incident Metabolic Syndrome. *PLoS One* **11**, e0168253, doi:10.1371/journal.pone.0168253 (2016).

70 Henninger, J., Hammarstedt, A., Rawshani, A. & Eliasson, B. Metabolic predictors of impaired glucose tolerance and type 2 diabetes in a predisposed population--A prospective cohort study. *BMC Endocr Disord* **15**, 51, doi:10.1186/s12902-015-0048-8 (2015).

71 Adams, S. H. *et al.* Plasma acylcarnitine profiles suggest incomplete long-chain fatty acid beta-oxidation and altered tricarboxylic acid cycle activity in type 2 diabetic African-American women. *J Nutr* **139**, 1073-1081, doi:10.3945/jn.108.103754 (2009).

72 Moore, S. C. *et al.* Human metabolic correlates of body mass index. *Metabolomics* **10**, 259-269, doi:10.1007/s11306-013-0574-1 (2014).

73 Menni, C. *et al.* Metabolomic identification of a novel pathway of blood pressure regulation involving hexadecanedioate. *Hypertension* **66**, 422-429, doi:10.1161/HYPERTENSIONAHA.115.05544 (2015).

74 Sha, W. *et al.* Metabolomic profiling can predict which humans will develop liver dysfunction when deprived of dietary choline. *FASEB J* **24**, 2962-2975, doi:10.1096/fj.09-154054 (2010).

75 Azuma, K. *et al.* Effects of Oral Administration of Chitin Nanofiber on Plasma Metabolites and Gut Microorganisms. *Int J Mol Sci* **16**, 21931-21949, doi:10.3390/ijms160921931 (2015).

76 Jang, C. *et al.* A branched-chain amino acid metabolite drives vascular fatty acid transport and causes insulin resistance. *Nat Med* **22**, 421-426, doi:10.1038/nm.4057 (2016).

77 Karaman Iu, K., Lobanova, E. G. & Iubitskaia, N. S. [Disturbed synthesis of eicosanoids in patients with metabolic syndrome]. *Klin Med (Mosk)* **88**, 46-49 (2010).

78 Gao, X. *et al.* Serum metabolic biomarkers distinguish metabolically healthy peripherally obese from unhealthy centrally obese individuals. *Nutr Metab (Lond)* **13**, 33, doi:10.1186/s12986-016-0095-9 (2016).

79 Ardiansyah *et al.* Novel effect of adenosine 5'-monophosphate on ameliorating hypertension and the metabolism of lipids and glucose in stroke-prone spontaneously hypertensive rats. *J Agric Food Chem* **59**, 13238-13245, doi:10.1021/jf203237c (2011).

80 Wang, X. H., Li, C. Y., Muhammad, I. & Zhang, X. Y. Fatty acid composition in serum correlates with that in the liver and non-alcoholic fatty liver disease activity scores in mice fed a high-fat diet. *Environ Toxicol Pharmacol* **44**, 140-150, doi:10.1016/j.etap.2016.04.009 (2016).

81 Park, M. Y. *et al.* Gut microbiota-associated bile acid deconjugation accelerates hepatic steatosis in ob/ob mice. *J Appl Microbiol* **121**, 800-810, doi:10.1111/jam.13158 (2016).

82 Garcia, A. *et al.* Helicobacter hepaticus--induced liver tumor promotion is associated with increased serum bile acid and a persistent microbial-induced immune response. *Cancer Res* **71**, 2529-2540, doi:10.1158/0008-5472.CAN-10-1975 (2011).

83 Kim, M. J. *et al.* Obesity-related metabolomic analysis of human subjects in black soybean peptide intervention study by ultraperformance liquid chromatography and quadrupole-time-of-flight mass spectrometry. *J Obes* **2013**, 874981, doi:10.1155/2013/874981 (2013).

84 Briot, A. *et al.* Endothelial NOTCH1 is suppressed by circulating lipids and antagonizes inflammation during atherosclerosis. *J Exp Med* **212**, 2147-2163, doi:10.1084/jem.20150603 (2015).

85 Guo, W. *et al.* Quantitative Metabolomic Profiling of Plasma, Urine, and Liver Extracts by (1)H NMR Spectroscopy Characterizes Different Stages of Atherosclerosis in Hamsters. *J Proteome Res* **15**, 3500-3510, doi:10.1021/acs.jproteome.6b00179 (2016).

86 Adam, A. C., Lie, K. K., Moren, M. & Skjaerven, K. H. High dietary arachidonic acid levels induce changes in complex lipids and immune-related eicosanoids and increase levels of oxidised metabolites in zebrafish (Danio rerio). *Br J Nutr* **117**, 1075-1085, doi:10.1017/S0007114517000903 (2017).

87 Gouveia-Figueira, S., Spath, J., Zivkovic, A. M. & Nording, M. L. Profiling the Oxylipin and Endocannabinoid Metabolome by UPLC-ESI-MS/MS in Human Plasma to Monitor Postprandial Inflammation. *PLoS One* **10**, e0132042, doi:10.1371/journal.pone.0132042 (2015).

88 Rossi, A. *et al.* Insulin-resistance in glycogen storage disease type Ia: linking carbohydrates and mitochondria? *J Inherit Metab Dis*, doi:10.1007/s10545-018-0149-4 (2018).

89 Dulin, J. N., Karoly, E. D., Wang, Y., Strobel, H. W. & Grill, R. J. Licofelone modulates neuroinflammation and attenuates mechanical hypersensitivity in the chronic phase of spinal cord injury. *J Neurosci* **33**, 652-664, doi:10.1523/JNEUROSCI.6128-11.2013 (2013).

90 Osmers, I. *et al.* Deletion of the G2A receptor fails to attenuate experimental autoimmune encephalomyelitis. *J Neuroimmunol* **207**, 18-23, doi:10.1016/j.jneuroim.2008.11.008 (2009).

91 Nieman, D. C. *et al.* Metabolomics approach to assessing plasma 13- and 9-hydroxy-octadecadienoic acid and linoleic acid metabolite responses to 75-km cycling. *Am J Physiol Regul Integr Comp Physiol* **307**, R68-74, doi:10.1152/ajpregu.00092.2014 (2014).

92 McDaniel, J. C., Massey, K. & Nicolaou, A. Fish oil supplementation alters levels of lipid mediators of inflammation in microenvironment of acute human wounds. *Wound Repair Regen* **19**, 189-200, doi:10.1111/j.1524-475X.2010.00659.x (2011).

93 Le, T. T., Ziemba, A., Urasaki, Y., Brotman, S. & Pizzorno, G. Label-free evaluation of hepatic microvesicular steatosis with multimodal coherent anti-Stokes Raman scattering microscopy. *PLoS One* **7**, e51092, doi:10.1371/journal.pone.0051092 (2012).

94 Ramsden, C. E. *et al.* Lowering dietary linoleic acid reduces bioactive oxidized linoleic acid metabolites in humans. *Prostaglandins Leukot Essent Fatty Acids* **87**, 135-141, doi:10.1016/j.plefa.2012.08.004 (2012).

95 Poreba, M. *et al.* Relationship between polyunsaturated fatty acid composition in serum phospholipids, systemic low-grade inflammation, and glycemic control in patients with type 2 diabetes and atherosclerotic cardiovascular disease. *Cardiovasc Diabetol* **17**, 29, doi:10.1186/s12933-018-0672-5 (2018).

96 Morin, B., Bubb, W. A., Davies, M. J., Dean, R. T. & Fu, S. 3-Hydroxylysine, a potential marker for studying radical-induced protein oxidation. *Chem Res Toxicol* **11**, 1265-1273, doi:10.1021/tx980118h (1998).

97 Du, F., Virtue, A., Wang, H. & Yang, X. F. Metabolomic analyses for atherosclerosis, diabetes, and obesity. *Biomark Res* **1**, 17, doi:10.1186/2050-7771-1-17 (2013).

98 Barone, M. *et al.* Influence of ursodeoxycholate-enriched diet on liver tumor growth in HBV transgenic mice. *Hepatology* **37**, 880-886, doi:10.1053/jhep.2003.50175 (2003).

99 Sankaranarayanan, K. *et al.* Gut Microbiome Diversity among Cheyenne and Arapaho Individuals from Western Oklahoma. *Curr Biol* **25**, 3161-3169, doi:10.1016/j.cub.2015.10.060 (2015).

100 Kaur, P. *et al.* Quantitative metabolomic and lipidomic profiling reveals aberrant amino acid metabolism in type 2 diabetes. *Mol Biosyst* **9**, 307-317, doi:10.1039/c2mb25384d (2013).

101 Zheng, J. S. *et al.* Serum metabolomics profiles in response to n-3 fatty acids in Chinese patients with type 2 diabetes: a double-blind randomised controlled trial. *Sci Rep* **6**, 29522, doi:10.1038/srep29522 (2016).

102 Boulange, C. L. *et al.* Early metabolic adaptation in C57BL/6 mice resistant to high fat diet induced weight gain involves an activation of mitochondrial oxidative pathways. *J Proteome Res* **12**, 1956-1968, doi:10.1021/pr400051s (2013).

103 Inoue, K., Kishida, K., Hirata, A., Funahashi, T. & Shimomura, I. Low serum eicosapentaenoic acid / arachidonic acid ratio in male subjects with visceral obesity. *Nutr Metab (Lond)* **10**, 25, doi:10.1186/1743-7075-10-25 (2013).

104 Murase, R. *et al.* Group X Secreted Phospholipase A2 Releases omega3 Polyunsaturated Fatty Acids, Suppresses Colitis, and Promotes Sperm Fertility. *J Biol Chem* **291**, 6895-6911, doi:10.1074/jbc.M116.715672 (2016).

105 Yuan, T. *et al.* [Association of red blood cell damage with arachidonic acid]. *Zhongguo Gu Shang* **29**, 179-183 (2016).

106 Horas, H. N. S. *et al.* Adrenic acid as an inflammation enhancer in non-alcoholic fatty liver disease. *Arch Biochem Biophys* **623-624**, 64-75, doi:10.1016/j.abb.2017.04.009 (2017).

107 Kaliannan, K. *et al.* Estrogen-mediated gut microbiome alterations influence sexual dimorphism in metabolic syndrome in mice. *Microbiome* **6**, 205, doi:10.1186/s40168-018-0587-0 (2018).

108 Kelder, T., Stroeve, J. H., Bijlsma, S., Radonjic, M. & Roeselers, G. Correlation network analysis reveals relationships between diet-induced changes in human gut microbiota and metabolic health. *Nutr Diabetes* **4**, e122, doi:10.1038/nutd.2014.18 (2014).

109 Robertson, R. C. *et al.* Maternal omega-3 fatty acids regulate offspring obesity through persistent modulation of gut microbiota. *Microbiome* **6**, 95, doi:10.1186/s40168-018-0476-6 (2018).

110 Pai, V. J., Wang, B., Li, X., Wu, L. & Kang, J. X. Transgenic mice convert carbohydrates to essential fatty acids. *PLoS One* **9**, e97637, doi:10.1371/journal.pone.0097637 (2014).

111 Kang, J. X., Wang, J., Wu, L. & Kang, Z. B. Transgenic mice: fat-1 mice convert n-6 to n-3 fatty acids. *Nature* **427**, 504, doi:10.1038/427504a (2004).

112 Kang, J. X. & Wang, J. A simplified method for analysis of polyunsaturated fatty acids. *BMC Biochem* **6**, 5, doi:10.1186/1471-2091-6-5 (2005).

113 Marvyn, P. M., Bradley, R. M., Mardian, E. B., Marks, K. A. & Duncan, R. E. Data on oxygen consumption rate, respiratory exchange ratio, and movement in C57BL/6J female mice on the third day of consuming a high-fat diet. *Data Brief* **7**, 472-475, doi:10.1016/j.dib.2016.02.066 (2016).

114 Jacobi, S. K. *et al.* Dietary Isomers of Sialyllactose Increase Ganglioside Sialic Acid Concentrations in the Corpus Callosum and Cerebellum and Modulate the Colonic Microbiota of Formula-Fed Piglets. *J Nutr* **146**, 200-208, doi:10.3945/jn.115.220152 (2016).

115 Edgar, R. C. UPARSE: highly accurate OTU sequences from microbial amplicon reads. *Nat Methods* **10**, 996-998, doi:10.1038/nmeth.2604 (2013).

116 Love, M. I., Huber, W. & Anders, S. Moderated estimation of fold change and dispersion for RNA-seq data with DESeq2. *Genome Biol* **15**, 550, doi:10.1186/s13059-014-0550-8 (2014).

117 McMurdie, P. J. & Holmes, S. Waste not, want not: why rarefying microbiome data is inadmissible. *PLoS Comput Biol* **10**, e1003531, doi:10.1371/journal.pcbi.1003531 (2014).

118 Chevalier, C. *et al.* Gut Microbiota Orchestrates Energy Homeostasis during Cold. *Cell* **163**, 1360-1374, doi:10.1016/j.cell.2015.11.004 (2015).

119 Segata, N. *et al.* Metagenomic biomarker discovery and explanation. *Genome Biol* **12**, R60, doi:10.1186/gb-2011-12-6-r60 (2011).

120 Dhariwal, A. *et al.* MicrobiomeAnalyst: a web-based tool for comprehensive statistical, visual and meta-analysis of microbiome data. *Nucleic Acids Res* **45**, W180-W188, doi:10.1093/nar/gkx295 (2017).

121 Stewart, C. J. *et al.* Gut microbiota of Type 1 diabetes patients with good glycaemic control and high physical fitness is similar to people without diabetes: an observational study. *Diabet Med* **34**, 127-134, doi:10.1111/dme.13140 (2017).

122 Sreekumar, A. *et al.* Metabolomic profiles delineate potential role for sarcosine in prostate cancer progression. *Nature* **457**, 910-914, doi:10.1038/nature07762 (2009).

123 Yuan, X. *et al.* Green Tea Liquid Consumption Alters the Human Intestinal and Oral Microbiome. *Mol Nutr Food Res* **62**, e1800178, doi:10.1002/mnfr.201800178 (2018).
