# Supplementary material for: Multi-omic analysis in transgenic mice implicates omega-6/omega-3 fatty acid imbalance as a risk factor for chronic disease
Source: Commun Biol. 2019 Jul 26;2:276. doi: 10.1038/s42003-019-0521-4 (PMC6659714; doi:10.1038/s42003-019-0521-4)
Supplement: Supplementary file 2 — Description of Additional Supplementary Files [file 42003_2019_521_MOESM2_ESM.docx]

**Description of additional supplementary items**

**Filename: Supplementary Data 1**

**Description:** The parameters [variable importance in the projection (VIP) values 1 or > 1.0] contributing to the multivariate partial least square-regression (PLS-R) model.
