# Supplementary material for: Multi-omic analysis in transgenic mice implicates omega-6/omega-3 fatty acid imbalance as a risk factor for chronic disease
Source: Commun Biol. 2019 Jul 26;2:276. doi: 10.1038/s42003-019-0521-4 (PMC6659714; doi:10.1038/s42003-019-0521-4)
Supplement: Supplementary file 3 — Supplementary Data 1 [file 42003_2019_521_MOESM3_ESM.docx]

**Supplementary Data.1.** Variable importance in project (VIP) scores, Related to Figure 4

| **Variable name** | **VIP** |
| --- | --- |
| Bacteria\|Proteobacteria\|Gammaproteobacteria\|Enterobacteriales\|Enterobacteriaceae\|unclassified\|unclassified\| | 3.254 |
| Bacteria\|Proteobacteria\|Gammaproteobacteria\|Enterobacteriales\|Enterobacteriaceae\|unclassified\|unclassified\| | 3.241 |
| Bacteria\|Proteobacteria\|Gammaproteobacteria\|Enterobacteriales\|Enterobacteriaceae\|unclassified\|unclassified\| | 3.236 |
| Bacteria\|Proteobacteria\|Gammaproteobacteria\|Enterobacteriales\|Enterobacteriaceae\|unclassified\|unclassified\| | 3.21 |
| Bacteria\|Proteobacteria\|Gammaproteobacteria\|Enterobacteriales\|Enterobacteriaceae\|unclassified\|unclassified\| | 3.199 |
| Bacteria\|Proteobacteria\|Gammaproteobacteria\|Enterobacteriales\|Enterobacteriaceae\|94otu9652\|97otu2810\| | 3.192 |
| Bacteria\|Proteobacteria\|Gammaproteobacteria\|Enterobacteriales\|Enterobacteriaceae\|unclassified\|unclassified\| | 3.138 |
| Bacteria\|Proteobacteria\|Gammaproteobacteria\|Enterobacteriales\|Enterobacteriaceae\|unclassified\|unclassified\| | 3.116 |
| Bacteria\|Proteobacteria\|unclassified\|unclassified\|unclassified\|unclassified\|unclassified\| | 3.023 |
| Bacteria\|Proteobacteria\|Deltaproteobacteria\|Desulfovibrionales\|Desulfovibrionaceae\|94otu12186\|unclassified\| | 3.02 |
| Bacteria\|Proteobacteria\|Gammaproteobacteria\|Enterobacteriales\|Enterobacteriaceae\|unclassified\|unclassified\| | 2.926 |
| Bacteria\|Proteobacteria\|Gammaproteobacteria\|Enterobacteriales\|Enterobacteriaceae\|unclassified\|unclassified\| | 2.868 |
| Bacteria\|Proteobacteria\|Deltaproteobacteria\|Desulfovibrionales\|Desulfovibrionaceae\|94otu12186\|unclassified\| | 2.755 |
| Bacteria\|Proteobacteria\|Gammaproteobacteria\|Enterobacteriales\|Enterobacteriaceae\|unclassified\|unclassified\| | 2.738 |
| Bacteria\|Proteobacteria\|Gammaproteobacteria\|Enterobacteriales\|Enterobacteriaceae\|unclassified\|unclassified\| | 2.725 |
| N-6/N-3 | 2.647 |
| Docosapentaenoate (n6 DPA; 22:5n6) | 2.644 |
| Bacteria\|Proteobacteria\|Gammaproteobacteria\|Enterobacteriales\|Enterobacteriaceae\|unclassified\|unclassified\| | 2.636 |
| Total N-6 | 2.627 |
| Adrenate (22:4n6) | 2.574 |
| Bacteria\|Proteobacteria\|Deltaproteobacteria\|Desulfovibrionales\|Desulfovibrionaceae\|94otu12186\|unclassified\| | 2.573 |
| N-acetylasparagine | 2.54 |
| Bilirubin (E,E) | 2.514 |
| 12-HETE | 2.471 |
| Bacteria\|Proteobacteria\|Gammaproteobacteria\|Enterobacteriales\|Enterobacteriaceae\|unclassified\|unclassified\| | 2.465 |
| Bacteria\|Proteobacteria\|Gammaproteobacteria\|Enterobacteriales\|Enterobacteriaceae\|unclassified\|unclassified\| | 2.45 |
| Bacteria\|Bacteroidetes\|Bacteroidia\|Bacteroidales\|91otu547\|94otu13803\|97otu65263\| | 2.446 |
| Arachidonate (20:4n6) | 2.342 |
| Aspartate | 2.327 |
| Bacteria\|Bacteroidetes\|Bacteroidia\|Bacteroidales\|S24-7\|unclassified\|unclassified\| | 2.307 |
| Bacteria\|Bacteroidetes\|Bacteroidia\|Bacteroidales\|91otu547\|94otu13803\|97otu65263\| | 2.303 |
| Glycine | 2.234 |
| Bacteria\|Bacteroidetes\|Bacteroidia\|Bacteroidales\|Bacteroidaceae\|Bacteroides\|unclassified\| | 2.217 |
| Eicosapentaenoate (EPA; 20:5n3) | 2.214 |
| Bacteria\|Firmicutes\|Clostridia\|Clostridiales\|Lachnospiraceae\|94otu1346\|97otu60654\| | 2.176 |
| Gamma-glutamylisoleucine | 2.168 |
| Bacteria\|Proteobacteria\|Alphaproteobacteria\|RF32\|91otu6104\|94otu24954\|unclassified\| | 2.158 |
| Bacteria\|Firmicutes\|Clostridia\|Clostridiales\|unclassified\|unclassified\|unclassified\| | 2.153 |
| Lactate | 2.15 |
| 1-(1-enyl-palmitoyl)-2-arachidonoyl-GPC (P-16:0/20:4)* | 2.141 |
| Bacteria\|Firmicutes\|Clostridia\|Clostridiales\|Ruminococcaceae\|Oscillospira\|unclassified\| | 2.118 |
| Acetylcarnitine | 2.108 |
| Bacteria\|Bacteroidetes\|Bacteroidia\|Bacteroidales\|unclassified\|unclassified\|unclassified\| | 2.094 |
| Pyridoxine (Vitamin B6) | 2.086 |
| Valine | 2.074 |
| Bacteria\|Bacteroidetes\|Bacteroidia\|Bacteroidales\|unclassified\|unclassified\|unclassified\| | 2.055 |
| Bacteria\|Firmicutes\|Clostridia\|Clostridiales\|91otu5541\|94otu22157\|unclassified\| | 2.054 |
| Alanine | 2.054 |
| 1-stearoyl-GPS (18:0)* | 2.051 |
| Bacteria\|Firmicutes\|Clostridia\|Clostridiales\|91otu1083\|94otu2069\|97otu98284\| | 2.046 |
| N-acetylalanine | 2.042 |
| Bacteria\|Proteobacteria\|Deltaproteobacteria\|Desulfovibrionales\|Desulfovibrionaceae\|94otu12186\|unclassified\| | 2.041 |
| Isoleucine | 2.034 |
| Bacteria\|Proteobacteria\|Epsilonproteobacteria\|Campylobacterales\|Helicobacteraceae\|94otu10429\|unclassified\| | 2.025 |
| Total N-3 | 2.024 |
| Kynurenine | 2.023 |
| 2-methylbutyrylcarnitine (C5) | 2.018 |
| Bacteria\|Bacteroidetes\|Bacteroidia\|Bacteroidales\|Bacteroidaceae\|Bacteroides\|unclassified\| | 2.017 |
| Bilirubin (Z,Z) | 2.005 |
| Stearidonate (18:4n3) | 1.999 |
| 4-hydroxyphenylpyruvate | 1.989 |
| 1-arachidonoyl-GPC (20:4n6)* | 1.983 |
| Docosapentaenoate (n3 DPA; 22:5n3) | 1.974 |
| Bacteria\|Bacteroidetes\|Bacteroidia\|Bacteroidales\|Bacteroidaceae\|Bacteroides\|unclassified\| | 1.964 |
| Bacteria\|Firmicutes\|Clostridia\|Clostridiales\|Ruminococcaceae\|unclassified\|unclassified\| | 1.963 |
| N-acetylglycine | 1.959 |
| Gamma-glutamylvaline | 1.952 |
| Bacteria\|Bacteroidetes\|Bacteroidia\|Bacteroidales\|Bacteroidaceae\|Bacteroides\|unclassified\| | 1.946 |
| Bacteria\|Bacteroidetes\|Bacteroidia\|Bacteroidales\|Bacteroidaceae\|Bacteroides\|unclassified\| | 1.942 |
| Bacteria\|Bacteroidetes\|Bacteroidia\|Bacteroidales\|Bacteroidaceae\|Bacteroides\|unclassified\| | 1.937 |
| Bacteria\|Bacteroidetes\|Bacteroidia\|Bacteroidales\|Bacteroidaceae\|Bacteroides\|97otu19740\| | 1.934 |
| Bacteria\|Firmicutes\|Clostridia\|Clostridiales\|Lachnospiraceae\|94otu11370\|97otu15132\| | 1.922 |
| Bacteria\|Firmicutes\|Clostridia\|Clostridiales\|91otu7271\|94otu3874\|97otu66543\| | 1.91 |
| Beta-guanidinopropanoate | 1.907 |
| Bacteria\|Bacteroidetes\|Bacteroidia\|Bacteroidales\|Bacteroidaceae\|Bacteroides\|unclassified\| | 1.898 |
| Bacteria\|Firmicutes\|Clostridia\|Clostridiales\|unclassified\|unclassified\|unclassified\| | 1.898 |
| Bacteria\|Bacteroidetes\|unclassified\|unclassified\|unclassified\|unclassified\|unclassified\| | 1.898 |
| Bacteria\|Proteobacteria\|Epsilonproteobacteria\|Campylobacterales\|Helicobacteraceae\|94otu10429\|unclassified\| | 1.898 |
| Bacteria\|Bacteroidetes\|Bacteroidia\|Bacteroidales\|Bacteroidaceae\|Bacteroides\|unclassified\| | 1.896 |
| Bacteria\|Firmicutes\|Clostridia\|Clostridiales\|Lachnospiraceae\|94otu24824\|97otu27233\| | 1.877 |
| Bacteria\|Bacteroidetes\|Bacteroidia\|Bacteroidales\|Bacteroidaceae\|Bacteroides\|unclassified\| | 1.876 |
| Bacteria\|Proteobacteria\|Deltaproteobacteria\|Desulfovibrionales\|Desulfovibrionaceae\|94otu12186\|97otu98262\| | 1.875 |
| Bacteria\|Proteobacteria\|Epsilonproteobacteria\|Campylobacterales\|Helicobacteraceae\|94otu10429\|unclassified\| | 1.871 |
| Bacteria\|Firmicutes\|Bacilli\|Lactobacillales\|Lactobacillaceae\|Lactobacillus\|unclassified\| | 1.871 |
| Succinate | 1.869 |
| Bacteria\|Actinobacteria\|Actinobacteria\|Bifidobacteriales\|Bifidobacteriaceae\|Bifidobacterium\|unclassified\| | 1.868 |
| Bacteria\|Bacteroidetes\|Bacteroidia\|Bacteroidales\|91otu547\|94otu13803\|97otu65263\| | 1.859 |
| Bacteria\|Firmicutes\|Bacilli\|Bacillales\|Staphylococcaceae\|Staphylococcus\|97otu72881\| | 1.854 |
| glycerophosphoglycerol | 1.853 |
| Bacteria\|Actinobacteria\|Coriobacteriia\|Coriobacteriales\|Coriobacteriaceae\|94otu19005\|unclassified\| | 1.853 |
| 1-stearoyl-2-arachidonoyl-GPC (18:0/20:4) | 1.852 |
| Threonine | 1.85 |
| Bacteria\|Bacteroidetes\|Bacteroidia\|Bacteroidales\|Bacteroidaceae\|Bacteroides\|unclassified\| | 1.843 |
| Bacteria\|Bacteroidetes\|Bacteroidia\|Bacteroidales\|Bacteroidaceae\|Bacteroides\|unclassified\| | 1.842 |
| Bacteria\|Bacteroidetes\|Bacteroidia\|Bacteroidales\|Bacteroidaceae\|Bacteroides\|unclassified\| | 1.831 |
| Bacteria\|Bacteroidetes\|Bacteroidia\|Bacteroidales\|Bacteroidaceae\|Bacteroides\|unclassified\| | 1.825 |
| Bacteria\|Bacteroidetes\|Bacteroidia\|Bacteroidales\|Bacteroidaceae\|Bacteroides\|unclassified\| | 1.82 |
| Leucine | 1.813 |
| 1-stearoyl-GPI (18:0) | 1.811 |
| Bacteria\|Firmicutes\|Bacilli\|Lactobacillales\|Streptococcaceae\|Lactococcus\|unclassified\| | 1.806 |
| Bacteria\|Bacteroidetes\|Bacteroidia\|Bacteroidales\|Bacteroidaceae\|Bacteroides\|unclassified\| | 1.804 |
| Serine | 1.801 |
| 1-(1-enyl-stearoyl)-2-arachidonoyl-GPE (P-18:0/20:4)* | 1.796 |
| Gamma-glutamylmethionine | 1.796 |
| Thromboxane B2 | 1.796 |
| Bacteria\|Bacteroidetes\|Bacteroidia\|Bacteroidales\|Bacteroidaceae\|Bacteroides\|unclassified\| | 1.795 |
| Indolelactate | 1.795 |
| Bacteria\|Firmicutes\|Clostridia\|Clostridiales\|[Mogibacteriaceae]\|94otu1330\|97otu1577\| | 1.787 |
| Bacteria\|Firmicutes\|Clostridia\|Clostridiales\|Ruminococcaceae\|Oscillospira\|97otu13166\| | 1.787 |
| 1-linoleoyl-2-arachidonoyl-GPC (18:2/20:4n6)* | 1.784 |
| Docosapentaenoate (n3 DPA; 22:5n3) | 1.778 |
| Phenylalanine | 1.776 |
| Bacteria\|Actinobacteria\|Actinobacteria\|Bifidobacteriales\|Bifidobacteriaceae\|Bifidobacterium\|unclassified\| | 1.776 |
| N-acetylthreonine | 1.775 |
| 13-HODE + 9-HODE | 1.774 |
| Bacteria\|Bacteroidetes\|Bacteroidia\|Bacteroidales\|91otu547\|94otu13803\|97otu65263\| | 1.77 |
| Bacteria\|Bacteroidetes\|Bacteroidia\|Bacteroidales\|Bacteroidaceae\|Bacteroides\|unclassified\| | 1.757 |
| Bacteria\|Proteobacteria\|Betaproteobacteria\|Burkholderiales\|Oxalobacteraceae\|Ralstonia\|97otu84368\| | 1.755 |
| Bacteria\|Bacteroidetes\|Bacteroidia\|Bacteroidales\|unclassified\|unclassified\|unclassified\| | 1.752 |
| Bacteria\|Verrucomicrobia\|Verrucomicrobiae\|Verrucomicrobiales\|Verrucomicrobiaceae\|Akkermansia\|muciniphila\| | 1.749 |
| Cis-4-decenoyl carnitine | 1.743 |
| Bacteria\|Firmicutes\|Clostridia\|Clostridiales\|unclassified\|unclassified\|unclassified\| | 1.735 |
| Tyrosine | 1.725 |
| Bacteria\|Bacteroidetes\|Bacteroidia\|Bacteroidales\|Bacteroidaceae\|Bacteroides\|unclassified\| | 1.722 |
| Bacteria\|Bacteroidetes\|Bacteroidia\|Bacteroidales\|[Odoribacteraceae]\|Odoribacter\|97otu18999\| | 1.719 |
| Bacteria\|Bacteroidetes\|Bacteroidia\|Bacteroidales\|Bacteroidaceae\|Bacteroides\|unclassified\| | 1.717 |
| gamma-glutamylleucine | 1.716 |
| Bacteria\|Firmicutes\|Clostridia\|Clostridiales\|Ruminococcaceae\|unclassified\|unclassified\| | 1.713 |
| Gamma-glutamylglutamate | 1.712 |
| Isovalerylcarnitine | 1.712 |
| Bacteria\|Proteobacteria\|Betaproteobacteria\|Burkholderiales\|Oxalobacteraceae\|unclassified\|unclassified\| | 1.705 |
| N-acetylhistidine | 1.705 |
| Bacteria\|Bacteroidetes\|Bacteroidia\|Bacteroidales\|Bacteroidaceae\|Bacteroides\|unclassified\| | 1.705 |
| Orotidine | 1.704 |
| Bacteria\|Firmicutes\|Clostridia\|Clostridiales\|Christensenellaceae\|94otu9537\|97otu75732\| | 1.701 |
| 1-stearoyl-2-arachidonoyl-GPE (18:0/20:4) | 1.697 |
| Bacteria\|Firmicutes\|Clostridia\|Clostridiales\|unclassified\|unclassified\|unclassified\| | 1.696 |
| Bacteria\|Bacteroidetes\|Bacteroidia\|Bacteroidales\|S24-7\|94otu17548\|97otu20731\| | 1.694 |
| Bacteria\|Firmicutes\|Clostridia\|Clostridiales\|unclassified\|unclassified\|unclassified\| | 1.683 |
| 1-palmitoyl-2-arachidonoyl-GPC (16:0/20:4) | 1.668 |
| Bacteria\|Bacteroidetes\|Bacteroidia\|Bacteroidales\|S24-7\|94otu4147\|97otu4768\| | 1.664 |
| Bacteria\|Bacteroidetes\|Bacteroidia\|Bacteroidales\|unclassified\|unclassified\|unclassified\| | 1.657 |
| Bacteria\|Bacteroidetes\|Bacteroidia\|Bacteroidales\|S24-7\|unclassified\|unclassified\| | 1.653 |
| Bacteria\|Bacteroidetes\|Bacteroidia\|Bacteroidales\|91otu547\|94otu13803\|97otu65263\| | 1.652 |
| Hexanoylcarnitine | 1.65 |
| Eicosapentaenoate (EPA; 20:5n3) | 1.646 |
| Spermidine | 1.645 |
| Bacteria\|Actinobacteria\|Actinobacteria\|Bifidobacteriales\|Bifidobacteriaceae\|Bifidobacterium\|unclassified\| | 1.644 |
| Bacteria\|Firmicutes\|Clostridia\|Clostridiales\|Ruminococcaceae\|94otu9261\|97otu8033\| | 1.637 |
| Methionine | 1.636 |
| Bacteria\|Firmicutes\|Clostridia\|Clostridiales\|unclassified\|unclassified\|unclassified\| | 1.634 |
| Bacteria\|Firmicutes\|Clostridia\|Clostridiales\|Ruminococcaceae\|Oscillospira\|unclassified\| | 1.632 |
| Bacteria\|Proteobacteria\|Deltaproteobacteria\|Desulfovibrionales\|Desulfovibrionaceae\|Desulfovibrio\|97otu16043\| | 1.629 |
| Bacteria\|Proteobacteria\|Gammaproteobacteria\|Enterobacteriales\|Enterobacteriaceae\|unclassified\|unclassified\| | 1.628 |
| Bacteria\|Proteobacteria\|Alphaproteobacteria\|RF32\|91otu6104\|94otu43320\|97otu48840\| | 1.622 |
| Methylphosphate | 1.621 |
| Bacteria\|Bacteroidetes\|Bacteroidia\|Bacteroidales\|91otu547\|94otu13803\|97otu65263\| | 1.62 |
| Bacteria\|Proteobacteria\|Deltaproteobacteria\|Desulfovibrionales\|Desulfovibrionaceae\|unclassified\|unclassified\| | 1.62 |
| 1-arachidonoyl-GPE (20:4n6) | 1.617 |
| 3-methyl-2-oxobutyrate | 1.616 |
| Bacteria\|Proteobacteria\|unclassified\|unclassified\|unclassified\|unclassified\|unclassified\| | 1.611 |
| gamma-glutamylphenylalanine | 1.604 |
| Bacteria\|Firmicutes\|Clostridia\|Clostridiales\|unclassified\|unclassified\|unclassified\| | 1.604 |
| Bacteria\|Firmicutes\|Clostridia\|Clostridiales\|unclassified\|unclassified\|unclassified\| | 1.595 |
| 2-stearoyl-GPE (18:0) | 1.594 |
| 12-HEPE | 1.59 |
| Gamma-glutamylglycine | 1.587 |
| N-acetylarginine | 1.586 |
| Trimethylamine N-oxide | 1.585 |
| Glycerophosphoinositol | 1.583 |
| 2-hydroxymyristate | 1.58 |
| 2'-deoxycytidine | 1.58 |
| Bacteria\|Bacteroidetes\|Bacteroidia\|Bacteroidales\|Bacteroidaceae\|Bacteroides\|unclassified\| | 1.576 |
| Cystine | 1.575 |
| N-acetylglutamine | 1.574 |
| Bacteria\|Verrucomicrobia\|Verrucomicrobiae\|Verrucomicrobiales\|Verrucomicrobiaceae\|Akkermansia\|muciniphila\| | 1.574 |
| 1-arachidonoyl-GPE (20:4n6)* | 1.574 |
| 1-arachidonoyl-GPA (20:4) | 1.571 |
| Bacteria\|Bacteroidetes\|Bacteroidia\|Bacteroidales\|Bacteroidaceae\|Bacteroides\|unclassified\| | 1.563 |
| Bacteria\|Firmicutes\|Clostridia\|Clostridiales\|91otu4244\|94otu28754\|97otu31668\| | 1.563 |
| Bacteria\|Bacteroidetes\|Bacteroidia\|Bacteroidales\|Bacteroidaceae\|Bacteroides\|unclassified\| | 1.563 |
| Bacteria\|Bacteroidetes\|Bacteroidia\|Bacteroidales\|Bacteroidaceae\|Bacteroides\|unclassified\| | 1.561 |
| Bacteria\|Firmicutes\|Clostridia\|Clostridiales\|Ruminococcaceae\|Ruminococcus\|unclassified\| | 1.558 |
| Tyrosylglycine | 1.556 |
| Glutamate | 1.554 |
| 2-hydroxybutyrate/2-hydroxyisobutyrate | 1.551 |
| Bacteria\|Firmicutes\|Clostridia\|Clostridiales\|Lachnospiraceae\|unclassified\|unclassified\| | 1.551 |
| Sphingosine | 1.545 |
| Proline | 1.543 |
| Bacteria\|Firmicutes\|Clostridia\|Clostridiales\|91otu5137\|94otu11136\|97otu26564\| | 1.541 |
| Bacteria\|Firmicutes\|Clostridia\|Clostridiales\|91otu4244\|94otu28754\|97otu70935\| | 1.541 |
| Bacteria\|Bacteroidetes\|Bacteroidia\|Bacteroidales\|S24-7\|94otu10608\|97otu12190\| | 1.541 |
| Bacteria\|Firmicutes\|Clostridia\|Clostridiales\|Lachnospiraceae\|94otu8753\|97otu2949\| | 1.541 |
| Bacteria\|Bacteroidetes\|Bacteroidia\|Bacteroidales\|S24-7\|94otu14847\|97otu16507\| | 1.541 |
| Bacteria\|Firmicutes\|Clostridia\|Clostridiales\|Ruminococcaceae\|unclassified\|unclassified\| | 1.541 |
| Bacteria\|Proteobacteria\|unclassified\|unclassified\|unclassified\|unclassified\|unclassified\| | 1.541 |
| Bacteria\|Firmicutes\|Clostridia\|Clostridiales\|Ruminococcaceae\|94otu9261\|97otu29263\| | 1.541 |
| Bacteria\|Firmicutes\|Clostridia\|Clostridiales\|91otu5137\|unclassified\|unclassified\| | 1.541 |
| Bacteria\|Bacteroidetes\|Bacteroidia\|Bacteroidales\|Bacteroidaceae\|Bacteroides\|caccae\| | 1.541 |
| Bacteria\|Firmicutes\|Clostridia\|Clostridiales\|unclassified\|unclassified\|unclassified\| | 1.541 |
| Bacteria\|Proteobacteria\|unclassified\|unclassified\|unclassified\|unclassified\|unclassified\| | 1.541 |
| Bacteria\|Proteobacteria\|Deltaproteobacteria\|Desulfovibrionales\|Desulfovibrionaceae\|Desulfovibrio\|unclassified\| | 1.541 |
| Bacteria\|Bacteroidetes\|Bacteroidia\|Bacteroidales\|Bacteroidaceae\|Bacteroides\|unclassified\| | 1.541 |
| Bacteria\|Actinobacteria\|Coriobacteriia\|Coriobacteriales\|Coriobacteriaceae\|Adlercreutzia\|unclassified\| | 1.538 |
| Cytidine 5'-monophosphate (5'-CMP) | 1.535 |
| Bacteria\|Firmicutes\|Erysipelotrichi\|Erysipelotrichales\|Erysipelotrichaceae\|Allobaculum\|97otu11096\| | 1.529 |
| Bacteria\|Firmicutes\|Bacilli\|Turicibacterales\|Turicibacteraceae\|Turicibacter\|97otu9642\| | 1.528 |
| Bacteria\|Bacteroidetes\|Bacteroidia\|Bacteroidales\|unclassified\|unclassified\|unclassified\| | 1.521 |
| Phenylpyruvate | 1.52 |
| Dihomo-linoleate (20:2n6) | 1.519 |
| Tryptophan | 1.518 |
| Bacteria\|Firmicutes\|Clostridia\|Clostridiales\|Ruminococcaceae\|unclassified\|unclassified\| | 1.517 |
| Histidine | 1.507 |
| Laurylcarnitine | 1.504 |
| Cis-urocanate | 1.504 |
| Bacteria\|Proteobacteria\|Deltaproteobacteria\|Desulfovibrionales\|Desulfovibrionaceae\|94otu12186\|unclassified\| | 1.504 |
| Bacteria\|Bacteroidetes\|Bacteroidia\|Bacteroidales\|S24-7\|94otu16997\|97otu114\| | 1.503 |
| Bacteria\|Proteobacteria\|Deltaproteobacteria\|Desulfovibrionales\|Desulfovibrionaceae\|94otu12186\|unclassified\| | 1.501 |
| Bacteria\|Bacteroidetes\|Bacteroidia\|Bacteroidales\|S24-7\|unclassified\|unclassified\| | 1.501 |
| Bacteria\|Firmicutes\|Clostridia\|Clostridiales\|91otu10708\|94otu3472\|97otu75254\| | 1.497 |
| Pantothenate | 1.497 |
| 1-palmitoyl-2-linoleoyl-GPE (16:0/18:2) | 1.495 |
| Linoleoylcarnitine | 1.493 |
| Bacteria\|Bacteroidetes\|Bacteroidia\|Bacteroidales\|Porphyromonadaceae\|Parabacteroides\|distasonis\| | 1.492 |
| 1-stearoyl-GPE (18:0) | 1.491 |
| 3-hydroxydecanoate | 1.485 |
| N1-Methyl-2-pyridone-5-carboxamide | 1.485 |
| Bacteria\|Proteobacteria\|Deltaproteobacteria\|Desulfovibrionales\|Desulfovibrionaceae\|94otu12186\|unclassified\| | 1.483 |
| N-acetylglutamate | 1.479 |
| Bacteria\|Firmicutes\|Erysipelotrichi\|Erysipelotrichales\|Erysipelotrichaceae\|94otu35801\|97otu58149\| | 1.478 |
| Bacteria\|Firmicutes\|Clostridia\|Clostridiales\|Ruminococcaceae\|Oscillospira\|unclassified\| | 1.478 |
| Octadecanedioate | 1.477 |
| Bacteria\|Firmicutes\|Clostridia\|Clostridiales\|Lachnospiraceae\|94otu11775\|97otu67608\| | 1.469 |
| Bacteria\|Firmicutes\|Clostridia\|Clostridiales\|Ruminococcaceae\|Oscillospira\|unclassified\| | 1.464 |
| Stearate (18:0) | 1.462 |
| Carnitine | 1.462 |
| Ribose | 1.461 |
| Bacteria\|Firmicutes\|Clostridia\|Clostridiales\|Ruminococcaceae\|Oscillospira\|unclassified\| | 1.46 |
| Bacteria\|Firmicutes\|Clostridia\|Clostridiales\|Lachnospiraceae\|94otu12084\|97otu9896\| | 1.452 |
| Bacteria\|Proteobacteria\|Deltaproteobacteria\|Desulfovibrionales\|Desulfovibrionaceae\|unclassified\|unclassified\| | 1.45 |
| Bacteria\|Bacteroidetes\|Bacteroidia\|Bacteroidales\|Bacteroidaceae\|Bacteroides\|unclassified\| | 1.447 |
| Alpha-muricholate | 1.446 |
| Myristoylcarnitine | 1.443 |
| Bacteria\|Bacteroidetes\|Bacteroidia\|Bacteroidales\|S24-7\|unclassified\|unclassified\| | 1.442 |
| 1-arachidonoyl-GPC (20:4n6)* | 1.439 |
| Alpha-CEHC | 1.437 |
| 1-arachidonoyl-GPI (20:4)* | 1.434 |
| Octadecanedioate | 1.434 |
| Bacteria\|Firmicutes\|Clostridia\|Clostridiales\|unclassified\|unclassified\|unclassified\| | 1.433 |
| Bacteria\|Firmicutes\|Clostridia\|Clostridiales\|unclassified\|unclassified\|unclassified\| | 1.431 |
| N-acetylserine | 1.43 |
| Bacteria\|Bacteroidetes\|Bacteroidia\|Bacteroidales\|Porphyromonadaceae\|Parabacteroides\|unclassified\| | 1.425 |
| Octanoylcarnitine | 1.424 |
| riboflavin (Vitamin B2) | 1.421 |
| Decanoylcarnitine | 1.419 |
| Bacteria\|Bacteroidetes\|Bacteroidia\|Bacteroidales\|S24-7\|94otu9699\|97otu21995\| | 1.417 |
| 4-hydroxybenzoate | 1.416 |
| Bacteria\|Firmicutes\|Clostridia\|Clostridiales\|Clostridiaceae\|94otu42703\|97otu48077\| | 1.413 |
| Undecanedioate | 1.405 |
| 2'-deoxyguanosine | 1.404 |
| Bacteria\|Firmicutes\|Clostridia\|Clostridiales\|Ruminococcaceae\|Oscillospira\|unclassified\| | 1.402 |
| Stearoylcarnitine | 1.402 |
| Azelate (nonanedioate) | 1.398 |
| 3-methyl-2-oxovalerate | 1.395 |
| Gamma-glutamylglutamine | 1.393 |
| Bacteria\|Firmicutes\|Clostridia\|Clostridiales\|Lachnospiraceae\|94otu1109\|97otu1318\| | 1.393 |
| Bacteria\|Firmicutes\|Clostridia\|Clostridiales\|Ruminococcaceae\|94otu37050\|97otu74343\| | 1.39 |
| Taurine | 1.388 |
| Bacteria\|Firmicutes\|Clostridia\|Clostridiales\|Ruminococcaceae\|Ruminococcus\|97otu19391\| | 1.387 |
| Bacteria\|Firmicutes\|Bacilli\|Lactobacillales\|Lactobacillaceae\|Lactobacillus\|unclassified\| | 1.387 |
| Bacteria\|Bacteroidetes\|Bacteroidia\|Bacteroidales\|S24-7\|94otu1086\|97otu86044\| | 1.385 |
| Hexadecanedioate | 1.385 |
| 1-oleoyl-2-linoleoyl-GPE (18:1/18:2)* | 1.385 |
| Bacteria\|Proteobacteria\|Epsilonproteobacteria\|Campylobacterales\|Helicobacteraceae\|94otu10429\|unclassified\| | 1.38 |
| Caproate (6:0) | 1.38 |
| Cytidine | 1.378 |
| Sphingosine | 1.371 |
| Bacteria\|Proteobacteria\|Gammaproteobacteria\|Enterobacteriales\|Enterobacteriaceae\|unclassified\|unclassified\| | 1.371 |
| 1-(1-enyl-palmitoyl)-2-arachidonoyl-GPE (P-16:0/20:4)* | 1.369 |
| Bacteria\|Firmicutes\|Clostridia\|Clostridiales\|unclassified\|unclassified\|unclassified\| | 1.366 |
| 1-stearoyl-GPC (18:0) | 1.366 |
| Benzoylcarnitine* | 1.365 |
| Bacteria\|Firmicutes\|Clostridia\|Clostridiales\|Lachnospiraceae\|Dorea\|97otu90286\| | 1.365 |
| Bacteria\|Bacteroidetes\|Bacteroidia\|Bacteroidales\|unclassified\|unclassified\|unclassified\| | 1.363 |
| N-acetyl-aspartyl-glutamate (NAAG) | 1.361 |
| Bacteria\|Bacteroidetes\|Bacteroidia\|Bacteroidales\|Bacteroidaceae\|Bacteroides\|unclassified\| | 1.358 |
| Bacteria\|Proteobacteria\|Deltaproteobacteria\|Desulfovibrionales\|Desulfovibrionaceae\|94otu9549\|97otu11118\| | 1.353 |
| Arabitol/xylitol | 1.353 |
| Bacteria\|Firmicutes\|Clostridia\|Clostridiales\|unclassified\|unclassified\|unclassified\| | 1.349 |
| 1-(1-enyl-stearoyl)-2-arachidonoyl-GPE (P-18:0/20:4)* | 1.347 |
| 3-hydroxyoctanoate | 1.345 |
| Bacteria\|Proteobacteria\|Deltaproteobacteria\|Desulfovibrionales\|Desulfovibrionaceae\|94otu12186\|unclassified\| | 1.345 |
| Bacteria\|Firmicutes\|Clostridia\|Clostridiales\|Lachnospiraceae\|Blautia\|obeum\| | 1.343 |
| Bacteria\|Bacteroidetes\|Bacteroidia\|Bacteroidales\|Bacteroidaceae\|Bacteroides\|unclassified\| | 1.337 |
| 3-methyladipate | 1.336 |
| Bacteria\|Proteobacteria\|Epsilonproteobacteria\|Campylobacterales\|Helicobacteraceae\|94otu10429\|unclassified\| | 1.333 |
| N-acetylmuramate | 1.332 |
| Bacteria\|Firmicutes\|Clostridia\|Clostridiales\|Ruminococcaceae\|unclassified\|unclassified\| | 1.332 |
| Bacteria\|Bacteroidetes\|Bacteroidia\|Bacteroidales\|unclassified\|unclassified\|unclassified\| | 1.33 |
| Bacteria\|Firmicutes\|Clostridia\|Clostridiales\|Ruminococcaceae\|unclassified\|unclassified\| | 1.329 |
| Alpha-hydroxyisovalerate | 1.327 |
| Cysteine s-sulfate | 1.325 |
| N-acetyltaurine | 1.324 |
| 1-stearoyl-2-linoleoyl-GPE (18:0/18:2)* | 1.32 |
| Gamma-glutamyl-epsilon-lysine | 1.318 |
| 1-methylhistidine | 1.318 |
| 2'-deoxyadenosine | 1.318 |
| Oleoylcarnitine | 1.316 |
| 1-(3-aminopropyl)-2-pyrrolidone | 1.315 |
| Corticosterone | 1.315 |
| N-acetylproline | 1.315 |
| Serotonin | 1.312 |
| Cysteine sulfinic acid | 1.311 |
| Retinol (Vitamin A) | 1.309 |
| Guanine | 1.307 |
| Bacteria\|Proteobacteria\|Deltaproteobacteria\|Desulfovibrionales\|Desulfovibrionaceae\|94otu12186\|unclassified\| | 1.305 |
| N6-carbamoylthreonyladenosine | 1.302 |
| Daidzein | 1.301 |
| Dimethylarginine (SDMA + ADMA) | 1.3 |
| 1-(1-enyl-oleoyl)-GPE (P-18:1) | 1.296 |
| Bacteria\|Firmicutes\|Clostridia\|Clostridiales\|Ruminococcaceae\|94otu7573\|97otu56547\| | 1.295 |
| Bacteria\|Firmicutes\|Clostridia\|Clostridiales\|Ruminococcaceae\|94otu34753\|unclassified\| | 1.292 |
| Erythronate | 1.292 |
| Gulonic acid | 1.291 |
| 3-hydroxylaurate | 1.289 |
| 1-palmitoyl-2-arachidonoyl-GPE (16:0/20:4) | 1.287 |
| Glucuronate | 1.287 |
| Cholate | 1.286 |
| N-formylmethionine | 1.285 |
| Bacteria\|Firmicutes\|Clostridia\|Clostridiales\|Ruminococcaceae\|94otu11945\|97otu43722\| | 1.284 |
| Biliverdin | 1.284 |
| Bacteria\|Firmicutes\|Clostridia\|Clostridiales\|Ruminococcaceae\|Oscillospira\|unclassified\| | 1.283 |
| Bacteria\|Firmicutes\|Clostridia\|Clostridiales\|91otu7644\|94otu37290\|97otu88588\| | 1.282 |
| Bacteria\|Firmicutes\|Clostridia\|Clostridiales\|Lachnospiraceae\|unclassified\|unclassified\| | 1.282 |
| Bacteria\|Firmicutes\|Clostridia\|Clostridiales\|Ruminococcaceae\|Oscillospira\|97otu18136\| | 1.282 |
| Bacteria\|Firmicutes\|Clostridia\|Clostridiales\|Ruminococcaceae\|94otu32010\|97otu35454\| | 1.279 |
| Phosphate | 1.277 |
| 2'-deoxyuridine | 1.277 |
| Bacteria\|Firmicutes\|Clostridia\|Clostridiales\|[Mogibacteriaceae]\|94otu13888\|unclassified\| | 1.276 |
| Bacteria\|Firmicutes\|Clostridia\|Clostridiales\|unclassified\|unclassified\|unclassified\| | 1.269 |
| Bacteria\|Proteobacteria\|Deltaproteobacteria\|Desulfovibrionales\|Desulfovibrionaceae\|94otu12186\|97otu98262\| | 1.265 |
| 2-aminooctanoate | 1.265 |
| Bacteria\|Bacteroidetes\|Bacteroidia\|Bacteroidales\|S24-7\|unclassified\|unclassified\| | 1.265 |
| 3-hydroxymyristate | 1.264 |
| Bacteria\|Firmicutes\|Clostridia\|Clostridiales\|Ruminococcaceae\|unclassified\|unclassified\| | 1.263 |
| Bacteria\|Firmicutes\|Clostridia\|Clostridiales\|Ruminococcaceae\|94otu42758\|97otu55209\| | 1.263 |
| Bacteria\|Firmicutes\|Clostridia\|Clostridiales\|91otu6920\|94otu16496\|97otu9697\| | 1.262 |
| Bacteria\|Verrucomicrobia\|Verrucomicrobiae\|Verrucomicrobiales\|Verrucomicrobiaceae\|Akkermansia\|muciniphila\| | 1.262 |
| Bacteria\|Firmicutes\|Clostridia\|Clostridiales\|Ruminococcaceae\|Ruminococcus\|unclassified\| | 1.261 |
| N-acetylglutamine | 1.26 |
| Bacteria\|Bacteroidetes\|Bacteroidia\|Bacteroidales\|91otu547\|94otu13803\|97otu65263\| | 1.257 |
| 1-linoleoyl-GPE (18:2)* | 1.256 |
| Bacteria\|Firmicutes\|Clostridia\|Clostridiales\|Ruminococcaceae\|unclassified\|unclassified\| | 1.255 |
| Bacteria\|Firmicutes\|Clostridia\|Clostridiales\|Lachnospiraceae\|Coprococcus\|97otu48800\| | 1.253 |
| N2,N2-dimethylguanosine | 1.252 |
| Bacteria\|Firmicutes\|Clostridia\|Clostridiales\|Ruminococcaceae\|Oscillospira\|unclassified\| | 1.251 |
| Erucate (22:1n9) | 1.25 |
| Dihomo-linolenate (20:3n3 or n6) | 1.25 |
| Trans-urocanate | 1.242 |
| Ursodeoxycholate | 1.241 |
| Sebacate (decanedioate) | 1.24 |
| Bacteria\|Firmicutes\|Bacilli\|Lactobacillales\|Streptococcaceae\|Streptococcus\|unclassified\| | 1.24 |
| Bacteria\|Firmicutes\|Clostridia\|Clostridiales\|Ruminococcaceae\|Oscillospira\|unclassified\| | 1.24 |
| Bacteria\|Firmicutes\|Clostridia\|Clostridiales\|[Mogibacteriaceae]\|94otu17676\|unclassified\| | 1.239 |
| 3-hydroxybutyrylcarnitine (1) | 1.239 |
| Bacteria\|Firmicutes\|Clostridia\|Clostridiales\|91otu7644\|94otu37290\|97otu88588\| | 1.232 |
| 3b-hydroxy-5-cholenoic acid | 1.232 |
| Pipecolate | 1.231 |
| Bacteria\|Proteobacteria\|Deltaproteobacteria\|Desulfovibrionales\|Desulfovibrionaceae\|94otu12186\|unclassified\| | 1.231 |
| Bacteria\|Bacteroidetes\|Bacteroidia\|Bacteroidales\|Bacteroidaceae\|Bacteroides\|unclassified\| | 1.23 |
| Docosahexaenoate (DHA; 22:6n3) | 1.229 |
| Bacteria\|Bacteroidetes\|Bacteroidia\|Bacteroidales\|S24-7\|unclassified\|unclassified\| | 1.228 |
| Fumarate | 1.227 |
| Guanidinosuccinate | 1.226 |
| Phenylacetylglycine | 1.225 |
| 1-(1-enyl-palmitoyl)-GPC (P-16:0)* | 1.222 |
| Bacteria\|Bacteroidetes\|Bacteroidia\|Bacteroidales\|Bacteroidaceae\|Bacteroides\|97otu19740\| | 1.221 |
| Bacteria\|Firmicutes\|Bacilli\|Lactobacillales\|Streptococcaceae\|Streptococcus\|unclassified\| | 1.218 |
| 7-alpha-hydroxy-3-oxo-4-cholestenoate (7-Hoca) | 1.217 |
| Bacteria\|Proteobacteria\|Epsilonproteobacteria\|Campylobacterales\|Helicobacteraceae\|94otu10429\|unclassified\| | 1.215 |
| Bacteria\|Firmicutes\|Clostridia\|Clostridiales\|Ruminococcaceae\|unclassified\|unclassified\| | 1.21 |
| Pyridoxate | 1.208 |
| Bacteria\|Firmicutes\|Clostridia\|Clostridiales\|Ruminococcaceae\|Oscillospira\|unclassified\| | 1.206 |
| Sphinganine | 1.205 |
| 2-hydroxyphenylacetate | 1.203 |
| Uracil | 1.203 |
| Bacteria\|Firmicutes\|Clostridia\|Clostridiales\|Ruminococcaceae\|Oscillospira\|unclassified\| | 1.203 |
| Bacteria\|Bacteroidetes\|Bacteroidia\|Bacteroidales\|Bacteroidaceae\|Bacteroides\|unclassified\| | 1.202 |
| N-delta-acetylornithine | 1.201 |
| N-acetylhistidine | 1.2 |
| 4-guanidinobutanoate | 1.197 |
| Bacteria\|Proteobacteria\|Gammaproteobacteria\|Enterobacteriales\|Enterobacteriaceae\|unclassified\|unclassified\| | 1.197 |
| 1-palmitoyl-GPC (16:0) | 1.196 |
| Imidazole lactate | 1.196 |
| Bacteria\|Firmicutes\|Erysipelotrichi\|Erysipelotrichales\|Erysipelotrichaceae\|Coprobacillus\|97otu73651\| | 1.195 |
| Glutarylcarnitine (C5) | 1.193 |
| 1-docosahexaenoylglycerol (22:6) | 1.192 |
| Lithocholate | 1.192 |
| Bacteria\|Firmicutes\|Clostridia\|Clostridiales\|Ruminococcaceae\|Oscillospira\|unclassified\| | 1.191 |
| 1-palmitoyl-2-arachidonoyl-GPC (16:0/20:4) | 1.191 |
| Bacteria\|Firmicutes\|Clostridia\|Clostridiales\|Ruminococcaceae\|unclassified\|unclassified\| | 1.19 |
| Bacteria\|Firmicutes\|Clostridia\|Clostridiales\|Lachnospiraceae\|94otu18071\|97otu53110\| | 1.19 |
| Bacteria\|Bacteroidetes\|Bacteroidia\|Bacteroidales\|Bacteroidaceae\|Bacteroides\|unclassified\| | 1.188 |
| Bacteria\|Firmicutes\|Clostridia\|Clostridiales\|91otu5577\|94otu16623\|97otu36005\| | 1.186 |
| Bacteria\|Firmicutes\|Clostridia\|Clostridiales\|Clostridiaceae\|94otu972\|97otu1180\| | 1.185 |
| Laurate (12:0) | 1.182 |
| Bacteria\|Deferribacteres\|Deferribacteres\|Deferribacterales\|Deferribacteraceae\|Mucispirillum\|schaedleri\| | 1.181 |
| Genistein | 1.181 |
| 3-hydroxysebacate | 1.181 |
| 1-stearoyl-2-arachidonoyl-GPC (18:0/20:4) | 1.181 |
| Bacteria\|Firmicutes\|Clostridia\|Clostridiales\|91otu7810\|94otu39587\|97otu26382\| | 1.18 |
| 3-hydroxysebacate | 1.18 |
| Biopterin | 1.177 |
| Glucuronate | 1.176 |
| Bacteria\|Firmicutes\|Clostridia\|Clostridiales\|Ruminococcaceae\|94otu18905\|97otu20811\| | 1.175 |
| Bacteria\|Firmicutes\|Clostridia\|Clostridiales\|91otu9715\|94otu18730\|97otu20632\| | 1.174 |
| Bacteria\|Bacteroidetes\|Bacteroidia\|Bacteroidales\|Porphyromonadaceae\|Parabacteroides\|distasonis\| | 1.172 |
| Bacteria\|Firmicutes\|Clostridia\|Clostridiales\|Ruminococcaceae\|94otu14327\|97otu33760\| | 1.171 |
| Bacteria\|Firmicutes\|Clostridia\|Clostridiales\|Ruminococcaceae\|Oscillospira\|97otu6084\| | 1.17 |
| Bacteria\|Bacteroidetes\|Bacteroidia\|Bacteroidales\|S24-7\|unclassified\|unclassified\| | 1.169 |
| Bacteria\|Firmicutes\|Clostridia\|Clostridiales\|Veillonellaceae\|Dialister\|97otu8330\| | 1.166 |
| Malate | 1.164 |
| Bacteria\|Firmicutes\|Clostridia\|Clostridiales\|Ruminococcaceae\|Oscillospira\|97otu80633\| | 1.162 |
| azelate (nonanedioate) | 1.16 |
| Bacteria\|Firmicutes\|Clostridia\|Clostridiales\|91otu7180\|94otu15349\|97otu28277\| | 1.16 |
| dihomo-linolenate (20:3n3 or n6) | 1.156 |
| Glutamine | 1.155 |
| Bacteria\|Bacteroidetes\|Bacteroidia\|Bacteroidales\|unclassified\|unclassified\|unclassified\| | 1.15 |
| Eicosenoate (20:1) | 1.15 |
| N2-acetyllysine | 1.15 |
| Bacteria\|Firmicutes\|Clostridia\|Clostridiales\|Ruminococcaceae\|Ruminococcus\|97otu13721\| | 1.149 |
| phenol sulfate | 1.148 |
| 10-nonadecenoate (19:1n9) | 1.147 |
| Bacteria\|Firmicutes\|Clostridia\|Clostridiales\|Dehalobacteriaceae\|Dehalobacterium\|97otu86093\| | 1.147 |
| Bacteria\|Bacteroidetes\|Bacteroidia\|Bacteroidales\|S24-7\|94otu18682\|97otu20578\| | 1.147 |
| Malonylcarnitine | 1.147 |
| 1-oleoyl-GPC (18:1) | 1.146 |
| Tauro-alpha-muricholate | 1.145 |
| Coprostanol | 1.145 |
| N6-carbamoylthreonyladenosine | 1.145 |
| Bacteria\|Firmicutes\|Clostridia\|Clostridiales\|unclassified\|unclassified\|unclassified\| | 1.144 |
| 7-methylguanine | 1.144 |
| Aconitate [cis or trans] | 1.14 |
| Bacteria\|Firmicutes\|Clostridia\|Clostridiales\|91otu10082\|94otu27226\|97otu37977\| | 1.137 |
| Bacteria\|unclassified\|unclassified\|unclassified\|unclassified\|unclassified\|unclassified\| | 1.136 |
| Bacteria\|Firmicutes\|Clostridia\|Clostridiales\|unclassified\|unclassified\|unclassified\| | 1.136 |
| Bacteria\|Proteobacteria\|Deltaproteobacteria\|Desulfovibrionales\|Desulfovibrionaceae\|94otu12186\|unclassified\| | 1.136 |
| Beta-alanine | 1.135 |
| Docosahexaenoate (DHA; 22:6n3) | 1.135 |
| Suberate (octanedioate) | 1.135 |
| 5-hydroxylysine | 1.133 |
| Bacteria\|Proteobacteria\|Gammaproteobacteria\|Pasteurellales\|Pasteurellaceae\|Aggregatibacter\|pneumotropica\| | 1.132 |
| Erythritol | 1.131 |
| Bacteria\|Firmicutes\|Clostridia\|Clostridiales\|unclassified\|unclassified\|unclassified\| | 1.131 |
| Alpha-tocopherol acetate | 1.129 |
| Bacteria\|Firmicutes\|Clostridia\|Clostridiales\|Ruminococcaceae\|Ruminococcus\|97otu81310\| | 1.128 |
| 2-methylbutyrylcarnitine (C5) | 1.127 |
| Linoleoylcarnitine | 1.126 |
| Laurylcarnitine | 1.125 |
| Myristoylcarnitine | 1.125 |
| Hexadecanedioate | 1.125 |
| Isovalerylcarnitine | 1.124 |
| Bacteria\|Firmicutes\|Clostridia\|Clostridiales\|unclassified\|unclassified\|unclassified\| | 1.124 |
| lysine | 1.121 |
| Threonate | 1.121 |
| Ophthalmate | 1.12 |
| Trigonelline (N'-methylnicotinate) | 1.12 |
| Glycerate | 1.12 |
| Dihomo-linoleate (20:2n6) | 1.12 |
| Sphinganine-1-phosphate | 1.119 |
| Bacteria\|Firmicutes\|Clostridia\|Clostridiales\|Lachnospiraceae\|Coprococcus\|97otu17439\| | 1.119 |
| 2-methylbutyrylglycine | 1.118 |
| Bacteria\|Bacteroidetes\|Bacteroidia\|Bacteroidales\|Bacteroidaceae\|Bacteroides\|unclassified\| | 1.117 |
| Gamma-glutamylalanine | 1.117 |
| Hexanoylcarnitine | 1.116 |
| Bacteria\|Firmicutes\|Clostridia\|Clostridiales\|Christensenellaceae\|94otu32868\|97otu83827\| | 1.116 |
| N-acetyltaurine | 1.115 |
| Bacteria\|Firmicutes\|Clostridia\|Clostridiales\|unclassified\|unclassified\|unclassified\| | 1.114 |
| 3-hydroxymyristate | 1.114 |
| Bacteria\|Firmicutes\|Clostridia\|Clostridiales\|91otu6340\|94otu10119\|97otu87645\| | 1.113 |
| Palmitate (16:0) | 1.112 |
| Tauro-beta-muricholate | 1.112 |
| Bacteria\|Firmicutes\|Clostridia\|Clostridiales\|Ruminococcaceae\|Oscillospira\|unclassified\| | 1.112 |
| Cytosine | 1.11 |
| Bacteria\|Firmicutes\|Clostridia\|Clostridiales\|Ruminococcaceae\|94otu23362\|97otu25607\| | 1.107 |
| Bacteria\|Firmicutes\|Clostridia\|Clostridiales\|Ruminococcaceae\|Oscillospira\|unclassified\| | 1.106 |
| Bacteria\|Firmicutes\|Clostridia\|Clostridiales\|Peptococcaceae\|94otu7801\|97otu9437\| | 1.104 |
| Valerylglycine | 1.103 |
| Bacteria\|Bacteroidetes\|Bacteroidia\|Bacteroidales\|S24-7\|94otu1116\|97otu8254\| | 1.103 |
| Trimethylamine N-oxide | 1.102 |
| O-sulfo-L-tyrosine | 1.101 |
| Bacteria\|Firmicutes\|Clostridia\|Clostridiales\|Ruminococcaceae\|94otu18214\|97otu20080\| | 1.098 |
| Allantoic acid | 1.098 |
| Bacteria\|Verrucomicrobia\|Verrucomicrobiae\|Verrucomicrobiales\|Verrucomicrobiaceae\|Akkermansia\|muciniphila\| | 1.097 |
| Formiminoglutamate | 1.096 |
| Bacteria\|Firmicutes\|Clostridia\|Clostridiales\|unclassified\|unclassified\|unclassified\| | 1.096 |
| Bacteria\|Firmicutes\|Clostridia\|Clostridiales\|Ruminococcaceae\|94otu33268\|97otu5781\| | 1.096 |
| Malonylcarnitine | 1.096 |
| Caproate (6:0) | 1.094 |
| Palmitoylcholine | 1.094 |
| Bacteria\|Bacteroidetes\|Bacteroidia\|Bacteroidales\|Bacteroidaceae\|Bacteroides\|unclassified\| | 1.093 |
| 1-methyladenine | 1.092 |
| Isovalerylglycine | 1.091 |
| Bacteria\|Proteobacteria\|Deltaproteobacteria\|Desulfovibrionales\|Desulfovibrionaceae\|94otu12186\|unclassified\| | 1.09 |
| Stearoylcarnitine | 1.09 |
| Bacteria\|Bacteroidetes\|Bacteroidia\|Bacteroidales\|Porphyromonadaceae\|Parabacteroides\|unclassified\| | 1.089 |
| Bacteria\|Firmicutes\|Bacilli\|Lactobacillales\|Enterococcaceae\|Enterococcus\|unclassified\| | 1.089 |
| Bacteria\|Firmicutes\|Clostridia\|Clostridiales\|Ruminococcaceae\|unclassified\|unclassified\| | 1.086 |
| Decanoylcarnitine | 1.083 |
| Bacteria\|Actinobacteria\|Actinobacteria\|Bifidobacteriales\|Bifidobacteriaceae\|Bifidobacterium\|unclassified\| | 1.082 |
| Bacteria\|Bacteroidetes\|Bacteroidia\|Bacteroidales\|S24-7\|unclassified\|unclassified\| | 1.081 |
| Bacteria\|Firmicutes\|Clostridia\|Clostridiales\|Christensenellaceae\|94otu45968\|97otu98887\| | 1.08 |
| 2-hydroxydecanoate | 1.075 |
| Methionine sulfoxide | 1.073 |
| Myristoleoylcarnitine | 1.072 |
| Tartarate | 1.072 |
| 3-sulfo-L-alanine | 1.072 |
| Bacteria\|Bacteroidetes\|Bacteroidia\|Bacteroidales\|Bacteroidaceae\|Bacteroides\|unclassified\| | 1.071 |
| Deoxycarnitine | 1.07 |
| 3-methylcrotonylglycine | 1.07 |
| Bacteria\|Firmicutes\|Clostridia\|Clostridiales\|unclassified\|unclassified\|unclassified\| | 1.07 |
| trans-4-hydroxyproline | 1.07 |
| Palmitoylcarnitine | 1.07 |
| Bacteria\|Bacteroidetes\|Bacteroidia\|Bacteroidales\|S24-7\|unclassified\|unclassified\| | 1.07 |
| Bacteria\|Firmicutes\|Clostridia\|Clostridiales\|Ruminococcaceae\|Oscillospira\|unclassified\| | 1.069 |
| Myo-inositol | 1.068 |
| Bacteria\|Firmicutes\|Clostridia\|Clostridiales\|Ruminococcaceae\|94otu40139\|97otu91725\| | 1.068 |
| Bacteria\|Firmicutes\|Clostridia\|Clostridiales\|Ruminococcaceae\|Oscillospira\|unclassified\| | 1.067 |
| 1-palmitoyl-2-linoleoyl-glycerol (16:0/18:2)* | 1.066 |
| Suberoylcarnitine | 1.066 |
| Bacteria\|Bacteroidetes\|Bacteroidia\|Bacteroidales\|Bacteroidaceae\|Bacteroides\|unclassified\| | 1.065 |
| Bacteria\|Proteobacteria\|Deltaproteobacteria\|Desulfovibrionales\|Desulfovibrionaceae\|94otu12186\|unclassified\| | 1.064 |
| 1-(1-enyl-palmitoyl)-2-arachidonoyl-GPE (P-16:0/20:4)* | 1.063 |
| Retinol (Vitamin A) | 1.063 |
| 4-hydroxycinnamate sulfate | 1.062 |
| Chenodeoxycholate | 1.062 |
| Butyrylglycine | 1.062 |
| Bacteria\|Bacteroidetes\|Bacteroidia\|Bacteroidales\|unclassified\|unclassified\|unclassified\| | 1.061 |
| Bacteria\|Bacteroidetes\|Bacteroidia\|Bacteroidales\|Bacteroidaceae\|Bacteroides\|unclassified\| | 1.058 |
| 1-(1-enyl-stearoyl)-2-oleoyl-GPE (P-18:0/18:1) | 1.057 |
| Gulonic acid | 1.055 |
| Sphingosine 1-phosphate | 1.055 |
| Bacteria\|Proteobacteria\|Gammaproteobacteria\|Enterobacteriales\|Enterobacteriaceae\|unclassified\|unclassified\| | 1.054 |
| Bacteria\|Firmicutes\|Clostridia\|Clostridiales\|Lachnospiraceae\|unclassified\|unclassified\| | 1.053 |
| Bacteria\|Firmicutes\|Clostridia\|Clostridiales\|unclassified\|unclassified\|unclassified\| | 1.053 |
| Bacteria\|Actinobacteria\|Coriobacteriia\|Coriobacteriales\|Coriobacteriaceae\|Adlercreutzia\|97otu69766\| | 1.053 |
| 3-methylglutaconate | 1.053 |
| 2-hydroxypalmitate | 1.052 |
| Bacteria\|Bacteroidetes\|Bacteroidia\|Bacteroidales\|S24-7\|94otu24209\|97otu74956\| | 1.051 |
| Bacteria\|Cyanobacteria\|4C0d-2\|YS2\|unclassified\|unclassified\|unclassified\| | 1.051 |
| Bacteria\|Proteobacteria\|Epsilonproteobacteria\|Campylobacterales\|Helicobacteraceae\|unclassified\|unclassified\| | 1.051 |
| Bacteria\|Bacteroidetes\|Bacteroidia\|Bacteroidales\|S24-7\|unclassified\|unclassified\| | 1.051 |
| Bacteria\|Firmicutes\|Clostridia\|Clostridiales\|Ruminococcaceae\|Ruminococcus\|unclassified\| | 1.051 |
| Pyruvate | 1.05 |
| Adenine | 1.05 |
| N1,N12-diacetylspermine | 1.05 |
| Bacteria\|Tenericutes\|Mollicutes\|RF39\|91otu7931\|94otu33882\|97otu84940\| | 1.049 |
| S-methylmethionine | 1.048 |
| Urea | 1.048 |
| Bacteria\|Bacteroidetes\|Bacteroidia\|Bacteroidales\|S24-7\|94otu11032\|97otu53444\| | 1.047 |
| 15-methylpalmitate | 1.045 |
| 4-methyl-2-oxopentanoate | 1.044 |
| Choline | 1.043 |
| Bacteria\|Firmicutes\|Clostridia\|Clostridiales\|unclassified\|unclassified\|unclassified\| | 1.043 |
| Deoxycholate | 1.043 |
| Bacteria\|Bacteroidetes\|Bacteroidia\|Bacteroidales\|Rikenellaceae\|94otu9546\|97otu6719\| | 1.042 |
| Bacteria\|Firmicutes\|Clostridia\|Clostridiales\|unclassified\|unclassified\|unclassified\| | 1.042 |
| Bacteria\|Bacteroidetes\|Bacteroidia\|Bacteroidales\|unclassified\|unclassified\|unclassified\| | 1.041 |
| 1-(3-aminopropyl)-2-pyrrolidone | 1.04 |
| Sphinganine | 1.04 |
| Bacteria\|Proteobacteria\|Deltaproteobacteria\|Desulfovibrionales\|Desulfovibrionaceae\|Bilophila\|97otu11197\| | 1.036 |
| Bacteria\|Firmicutes\|Clostridia\|Clostridiales\|unclassified\|unclassified\|unclassified\| | 1.036 |
| N1-methyladenosine | 1.035 |
| 1-stearoyl-2-oleoyl-GPE (18:0/18:1) | 1.033 |
| Bacteria\|Firmicutes\|Clostridia\|Clostridiales\|Ruminococcaceae\|unclassified\|unclassified\| | 1.033 |
| 6-hydroxyindole sulfate | 1.032 |
| Leucine | 1.031 |
| 5-methylcytidine | 1.029 |
| Bacteria\|Firmicutes\|Bacilli\|Lactobacillales\|Lactobacillaceae\|Lactobacillus\|reuteri\| | 1.029 |
| Tartronate (hydroxymalonate) | 1.027 |
| Bacteria\|Bacteroidetes\|Bacteroidia\|Bacteroidales\|Bacteroidaceae\|Bacteroides\|acidifaciens\| | 1.026 |
| Bacteria\|Firmicutes\|Clostridia\|Clostridiales\|Ruminococcaceae\|Oscillospira\|97otu63987\| | 1.025 |
| Bacteria\|Firmicutes\|Clostridia\|Clostridiales\|unclassified\|unclassified\|unclassified\| | 1.021 |
| 5-methyluridine (ribothymidine) | 1.02 |
| 5-oxoproline | 1.02 |
| 3-hydroxypalmitate | 1.018 |
| Bacteria\|Bacteroidetes\|Bacteroidia\|Bacteroidales\|unclassified\|unclassified\|unclassified\| | 1.018 |
| Bacteria\|Firmicutes\|Clostridia\|Clostridiales\|unclassified\|unclassified\|unclassified\| | 1.017 |
| Bacteria\|Proteobacteria\|Deltaproteobacteria\|Desulfovibrionales\|Desulfovibrionaceae\|94otu12186\|unclassified\| | 1.017 |
| Butyrylcarnitine | 1.017 |
| Bacteria\|Firmicutes\|Clostridia\|Clostridiales\|91otu9120\|94otu17609\|97otu97455\| | 1.016 |
| Bacteria\|Bacteroidetes\|Bacteroidia\|Bacteroidales\|S24-7\|unclassified\|unclassified\| | 1.015 |
| 1-linoleoyl-GPC (18:2) | 1.015 |
| Lactosyl-N-palmitoyl-sphingosine | 1.015 |
| Margarate (17:0) | 1.014 |
| Beta-muricholate | 1.013 |
| N6-succinyladenosine | 1.013 |
| N-acetylasparagine | 1.012 |
| Bacteria\|Firmicutes\|Clostridia\|Clostridiales\|Ruminococcaceae\|Ruminococcus\|97otu82418\| | 1.012 |
| Chenodeoxycholate | 1.01 |
| 3-hydroxypalmitate | 1.008 |
| Isovalerate | 1.007 |
| Cystathionine | 1.007 |
| Orotidine | 1.006 |
| N1-Methyl-2-pyridone-5-carboxamide | 1.006 |
| Tryptophan betaine | 1.006 |
| 12,13-DiHOME | 1.005 |
| N-acetylphenylalanine | 1.005 |
| Pseudouridine | 1.005 |
| Nicotinamide riboside | 1.004 |
| Asparagine | 1.004 |
| Ribitol | 1.002 |
| Tetradecanedioate | 1.001 |
| N6,N6,N6-trimethyllysine | 1 |
| Taurocholenate sulfate | 1 |
| Bacteria\|Firmicutes\|Clostridia\|Clostridiales\|unclassified\|unclassified\|unclassified\| | 1 |
