# Supplementary material for: Multi-omic analysis in transgenic mice implicates omega-6/omega-3 fatty acid imbalance as a risk factor for chronic disease
Source: Commun Biol. 2019 Jul 26;2:276. doi: 10.1038/s42003-019-0521-4 (PMC6659714; doi:10.1038/s42003-019-0521-4)
Supplement: Supplementary file 4 — Reporting Summary [file 42003_2019_521_MOESM4_ESM.pdf]

nature research

Corresponding author(s): Jing X. Kang
Last updated by author(s): Jun 11, 2019

Reporting Summary

Nature Research wishes to improve the reproducibility of the work that we publish. This form provides structure for consistency and transparency in reporting. For further information on Nature Research policies, see Authors & Referees and the Editorial Policy Checklist.

Statistics

For all statistical analyses, confirm that the following items are present in the figure legend, table legend, main text, or Methods section.

n/a	Confirmed	
☐	☒	The exact sample size (*n*) for each experimental group/condition, given as a discrete number and unit of measurement
☐	☒	A statement on whether measurements were taken from distinct samples or whether the same sample was measured repeatedly
☐	☒	The statistical test(s) used AND whether they are one- or two-sided<br>*Only common tests should be described solely by name; describe more complex techniques in the Methods section.*
☐	☒	A description of all covariates tested
☐	☒	A description of any assumptions or corrections, such as tests of normality and adjustment for multiple comparisons
☐	☒	A full description of the statistical parameters including central tendency (e.g. means) or other basic estimates (e.g. regression coefficient) AND variation (e.g. standard deviation) or associated estimates of uncertainty (e.g. confidence intervals)
☐	☒	For null hypothesis testing, the test statistic (e.g. *F*, *t*, *r*) with confidence intervals, effect sizes, degrees of freedom and *P* value noted<br>*Give P values as exact values whenever suitable.*
☒	☐	For Bayesian analysis, information on the choice of priors and Markov chain Monte Carlo settings
☐	☒	For hierarchical and complex designs, identification of the appropriate level for tests and full reporting of outcomes
☐	☒	Estimates of effect sizes (e.g. Cohen's *d*, Pearson's *r*), indicating how they were calculated

Our web collection on statistics for biologists contains articles on many of the points above.

Software and code

Policy information about availability of computer code

Data collection	1) Metabolync Portal (https://portal.metabolon.com) for fecal and serum metabolomics 2) GC ChemStation Software (Agilent)) 3) SecondgenomeR package: 0.2.4 (full data analysis pipeline for Second Genome's Microbial Profiling Service) (https://www.secondgenome.com/platform/microbiome-technologies/16s-sequencing-community-analysis. 4) Secondgenome Phylogenetic Investigation of Communities by Reconstruction of Unobserved States (PICRUSt). 5) Xponent software (Luminex, Austin, TX). 6) PerkinElmer M1 integrator. 7) Perkin-Elmer Fluorospectrophotometer. 8) LSM10, Zeiss immunofluorescence microscope.
Data analysis	1) GraphPad Prism version 6 &7.01. 2) XLSTAT (version 2017.6) including 3D plot. 3) PAST (version 2.17 & 3.11). 4) . SIMCA trial version 14.1. 5) Metabolync Portal (https://portal.metabolon.com). 6) GC ChemStation Software (Agilent). 7) SecondgenomeR package: 0.2.4 (full data analysis pipeline for Second Genome's Microbial Profiling Service) (https://www.secondgenome.com/platform/microbiome-technologies/16s-sequencing-community-analysis). 8) Secondgenome DESeq2 package. 9) Online galaxy server (https://huttenhower.sph.harvard.edu/galaxy/). 10) Secondgenome Phylogenetic Investigation of Communities by Reconstruction of Unobserved States (PICRUSt). 11) Xponent software (Luminex, Austin, TX). 12) PerkinElmer M1 integrator. 13) MicrobiomeAnalyst (https://www.microbiomeanalyst.ca/). 14) ImageJ software.

For manuscripts utilizing custom algorithms or software that are central to the research but not yet described in published literature, software must be made available to editors/reviewers. We strongly encourage code deposition in a community repository (e.g. GitHub). See the Nature Research guidelines for submitting code & software for further information.

Data

Policy information about availability of data

All manuscripts must include a data availability statement. This statement should provide the following information, where applicable:

- Accession codes, unique identifiers, or web links for publicly available datasets
- A list of figures that have associated raw data
- A description of any restrictions on data availability

Source data of main figure 1 panels (except for 1d, e, g, h and i), 16S sequencing details, OTU tables, raw data, taxonomy, FASTA files, KEGG and KO pathways and

abundance data collected using PICRUSt software and metadata for 16S rRNA gene sequence analysis performed by Secondgenome microbiome profiling company (EPAN15-0231), and global fecal and serum metabolome data (MGPT-01-15VW) (OrigScale, ScaledImpData, biochemical name and pathway IDs) performed by Metabolon Inc (https://www.metabolon.com/) in this study have been made publicly available in Figshare (https://figshare.com/s/89bd8826c65b96c7e197). Figures that have associated raw data are main figures 1-6, and Supplementary figures 1, 4 and 5. All the remaining data will be made available by the corresponding author upon reasonable request.

Field-specific reporting

Please select the one below that is the best fit for your research. If you are not sure, read the appropriate sections before making your selection.

☒ Life sciences ☐ Behavioural & social sciences ☐ Ecological, evolutionary & environmental sciences

For a reference copy of the document with all sections, see nature.com/documents/nr-reporting-summary-flat.pdf

Life sciences study design

All studies must disclose on these points even when the disclosure is negative.

Sample size

No statistical methods were used to predetermine sample sizes. Sample sizes were determined based on previous studies from our group and publications in the field.

Data exclusions

We did not have pre-established exclusion criteria. We followed the manufacture instructions to fulfill the requirements related to sample quality and quantity. Accordingly, we had to exclude two samples per group for the serum analysis of markers of metabolic endotoxemia and chronic low-grade inflammation because of excessive hemolysis while separating the serum from whole blood. In one group, n=7 for the serum TNF-α analysis because of a technical reason encountered with the instrument. For glucose tolerance test analysis, we excluded two samples per group as they were obtained from stressed animals.

Replication

A few experiments (body weight gain, glucose tolerance test, Oil Red O staining for fatty liver, markers of metabolic endotoxemia and chronic low-grade inflammation and qPCR-based targeted analysis of fecal bacteria) were replicated at least twice with reproducible results.

Randomization

Because our study used 3 different unique transgenic mouse models, we have chosen those mice expressed typical phenotypes determined using the tail omega-6 and omega-3 polyunsaturated fatty acid levels. All animals used were age and sex matched. Animals were randomly allocated to each group.

Blinding

The data collection was not blinded. Blinding was not possible as the investigators were also conducting the experiments and had to be aware of controls and treated groups.

Reporting for specific materials, systems and methods

We require information from authors about some types of materials, experimental systems and methods used in many studies. Here, indicate whether each material, system or method listed is relevant to your study. If you are not sure if a list item applies to your research, read the appropriate section before selecting a response.

Materials & experimental systems

n/a	Involved in the study	
☐	☒	Antibodies
☒	☐	Eukaryotic cell lines
☒	☐	Palaeontology
☐	☒	Animals and other organisms
☒	☐	Human research participants
☒	☐	Clinical data

Methods

n/a	Involved in the study	
☒	☐	ChIP-seq
☒	☐	Flow cytometry
☒	☐	MRI-based neuroimaging

Antibodies

Antibodies used

Primary antibodies for ZO-1 (GTX108592) and TLR4 (GTX125909) from GeneTex, San Antonio, TX, USA.

Validation

All antibodies used in this study are from commercial sources and have been validated by the vendors. Validation data are available on the manufacturer's website. ZO-1 Validation: http://www.genetex.com/ZO-1-antibody-GTX108592.html and http://www.genetex.com/Web/LandingPage/LandingPageView.aspx?n=216. Reference: Nat Methods. 2016 Oct;13(10):823-7.GeneTex website said that the GTX125909 is currently not offered. So we here provided a PMID (27183919) for an article used the same TLR4 antibody from GeneTex. Appropriate antibody dilutions were performed based on preliminary experiments.

Animals and other organisms

Policy information about studies involving animals; ARRIVE guidelines recommended for reporting animal research

Laboratory animals	Male FAT-2, FAT-1 and FAT-1+2 transgenic and wild-type mice used in this study were of a C57BL/6 background and bred and maintained under specific pathogen-free (SPF) conditions at the Massachusetts General Hospital (MGH) animal facility. The age of mice at the time of sample collection was mentioned in the manuscript.
Wild animals	The study did not involve wild animals.
Field-collected samples	The study did not involve samples collected from the field.
Ethics oversight	All animal protocols (Protocol #: 2010N000038) were reviewed and approved by the IACUC at MGH. Animals were sacrificed by the Animal Veterinary Medical Association (AVMA)-approved protocol.

Note that full information on the approval of the study protocol must also be provided in the manuscript.
